# Using computer simulation models to investigate the most promising microRNAs to improve muscle regeneration during ageing

**DOI:** 10.1038/s41598-017-12538-6

**Published:** 2017-09-26

**Authors:** Carole J. Proctor, Katarzyna Goljanek-Whysall

**Affiliations:** 1 MRC/Arthritis Research UK Centre for Musculoskeletal Ageing (CIMA), Institute of Cellular Medicine and Newcastle University Institute for Ageing, Newcastle University, Newcastle upon Tyne, UK; 2 MRC/Arthritis Research UK Centre for Musculoskeletal Ageing (CIMA), Department of Musculoskeletal Biology, Institute of Ageing and Chronic Disease, University of Liverpool, Liverpool, UK

**Keywords:** Computational models, miRNAs

## Abstract

MicroRNAs (miRNAs) regulate gene expression through interactions with target sites within mRNAs, leading to enhanced degradation of the mRNA or inhibition of translation. Skeletal muscle expresses many different miRNAs with important roles in adulthood myogenesis (regeneration) and myofibre hypertrophy and atrophy, processes associated with muscle ageing. However, the large number of miRNAs and their targets mean that a complex network of pathways exists, making it difficult to predict the effect of selected miRNAs on age-related muscle wasting. Computational modelling has the potential to aid this process as it is possible to combine models of individual miRNA:target interactions to form an integrated network. As yet, no models of these interactions in muscle exist. We created the first model of miRNA:target interactions in myogenesis based on experimental evidence of individual miRNAs which were next validated and used to make testable predictions. Our model confirms that miRNAs regulate key interactions during myogenesis and can act by promoting the switch between quiescent/proliferating/differentiating myoblasts and by maintaining the differentiation process. We propose that a threshold level of miR-1 acts in the initial switch to differentiation, with miR-181 keeping the switch on and miR-378 maintaining the differentiation and miR-143 inhibiting myogenesis.

## Introduction

Muscle homeostasis is perturbed in ageing leading to loss of muscle mass and function - sarcopenia. Muscle homeostasis depends on the balance between muscle hypertrophy and atrophy and successful muscle regeneration following injury. During ageing, muscle regeneration is defective and this is attributed, at least to some degree, to changes in satellite cell properties^1,2^. The mechanisms leading to this loss in homeostasis are not fully understood but it is hypothesised that changes in gene expression and dysregulation of cellular signalling pathways are key contributors. Recently, miRNAs have been shown to play important roles in muscle maintenance during adulthood and ageing^3–5^. Muscles express their own set of muscle-enriched miRNAs known as myomiRs: miR-1, -206, -208, -133, -486 and -499^6^. Satellite cells, adult muscle stem cells contributing to muscle regeneration, express different sets of miRNAs during quiescence and activation^1^ suggesting the role of miRNAs in regulating satellite cell homeostasis. Moreover, the expression profile of miRNAs in satellite cells and muscle is altered during exercise and ageing^3,5,7,8^. The formation of new muscle fibres and repair of injured myofibres (myogenesis) requires the differentiation of myogenic progenitors or satellite cells, respectively^9^ and several miRNAs have been shown to regulate myogenesis in adulthood, including miR-1, miR-206, miR-27, miR-378 and miR-181^4,10–12^.

We have shown that miR-1 and miR-206 down-regulate the expression of the transcription factor Pax3, which inhibits MyoD, a myogenic regulatory factor (MRF) that is essential for myogenesis to occur^13^. On the other hand, MyoD has been shown to upregulate the expression of many miRNAs, including miR-1^14^. Pax3/Pax7 are expressed in embryonic myogenic progenitor cells and in quiescent satellite cells, whereas MyoD expression is low in the undifferentiated progenitors. Upon activation of satellite cells/myogenic progenitors, the expression of Pax3 is downregulated, whereas MyoD expression is upregulated following satellite cell activation. The downregulation of Pax3/Pax7 expression is partly mediated by miR-1 and miR-206, which ensure complete silencing of the Pax genes to confer robustness to the timing of myogenesis in development^13^. In addition, miR-206 and miR-486 have been shown to induce myoblast differentiation by downregulating Pax7 expression^15^, and miR-27 inhibits Pax3 expression in satellite cells^16^. It has also been shown that Pax3 protein is regulated by monoubiquitination and degradation by the proteasome^17^. On the other hand, MyoD expression has been shown to be inhibited by Musculin (Msc) (also known as MyoR), expressed during early stages of myogenesis and down-regulated at later stages. Therefore, the balance between these two, as well as other myogenic factors such as Myf-5^18^, contributes to the regulation of early stages of myogenesis: commitment and differentiation. Moreover, the expression of miR-1 and miR-133 has been shown to decrease during skeletal muscle hypertrophy and ageing^7,19^.

Another miRNA, miR-181, is strongly upregulated during myogenesis and positively regulates myogenic differentiation through inhibiting Hox-A11, another antagonist of MyoD, expression^12^. miR-181 inhibits Hox-A11 at the protein level, which suggests that it inhibits translation of Hox-A11 mRNA^12^. miR-181 has also been shown to inhibit Sirt-1 expression at the protein level, which leads to a decrease in myotube size^20^. This may be partly mediated by Sirt-1 inhibition of FoxO3, a transcription factor that upregulates the muscle-specific E3 ligase Mafbx which in turn leads to an increase in protein degradation and muscle atrophy. Interestingly, MyoD has been shown to be one of the targets of Mafbx^21^. Sirt-1 has also been shown to promote cell proliferation by decreasing the expression of the cyclin-dependent kinase inhibitor, p21^22^ and to block the induction of autophagy-related genes leading to a decrease in proteolysis^23^. Therefore, miR-181 may inhibit Sirt-1-induced hypertrophy. Moreover, upregulation of miR-181 may also lead to inhibition of myoblast proliferation. miR-181a has been shown to be down-regulated in mice and human skeletal tissue with age^20^, therefore it may contribute to dysregulation of myogenesis with age.

During myogenesis, MyoD activation leads to upregulation of the expression of several miRNAs, including miR-378, an inhibitor of Msc^11^. miR-378 may provide robustness to myogenesis through inhibition of Msc expression during later stages of the differentiation process. During C2C12 differentiation, miR-378 expression is increased more than four-fold, with a slight increase on day 1, a larger increase on day 2 and no further increase up to day 4, suggesting its key role during early steps of myogenesis^11^. Interestingly, miR-378 is encoded within an intron of the Ppargc1b gene which encodes PGC-1β, a regulator of energy metabolism. As miR-378 is co-expressed with PGC-1β, it has been suggested to play a role in metabolism and known interactions with target genes has confirmed this (reviewed in^24^). miR-378a-5p has been shown to be down-regulated with age in men^7^. This study also showed that after exercise miR-378a-5p is downregulated in young but not in older people, suggesting a blunting effect of age on miR-378 expression in muscle following exercise. miR-378 and several other miRNAs also target the components of the insulin-like growth factor-1 (IGF-1) signalling pathway, resulting in loss of muscle homeostasis^7^. However, there have been no studies to date to show that miR-378a-3p, the strand investigated in other studies changes with age^3^.

A recent study identified miR-143–3p as a regulator of the IGF-binding protein-5 (Igfbp5), that regulates IGF signalling through binding IGF-1 and IGF-2, in primary myoblasts^25^. miR-143-3p expression is downregulated in satellite cells and muscle progenitors during ageing, and changes in miR-143:Igfbp5 interactions have been proposed to be a part of a compensatory mechanism aimed at improving the efficiency of myogenesis during ageing. Interestingly, treatment of myoblasts with the cytokine IL-6, which has been shown to be elevated in circulation during ageing^26^, led to lower expression of miR-143 and an increase in Igfbp5^25^. However, it is not yet clear how IL-6 leads to the reduction in miR-143 expression.

We have previously demonstrated the utility of computer simulation models to examine the effect of miRNAs in gene regulatory circuits (in submission). These models show how miRNAs affect target gene expression by different mechanisms such as positive feedback, negative feedback and positive feedforward loops. Each type of mechanism leads to different effects on the target genes and includes pulse-like responses, extended repression, delays in responses and fine-tuning. The aim of this study was to use computational modelling to increase our understanding of the mechanisms by which individual miRNA:target interactions affect the decline in muscle regeneration during ageing. In order to do this, we constructed network models of a subset of miRNAs that are known to be important in the maintenance of muscle homeostasis and are up- or down-regulated during ageing. To start modelling ageing-associated changes in the muscle, we chose the networks involving miR-1, miR-143, miR-181, and miR-378, miRNAs with roles in muscle homeostasis and expression of which is dysregulated in muscle and satellite cells during ageing.

## Results

### The miRNAs miR-1, miR-181, miR-378 and miR-143 affect myogenesis and are down-regulated in myogenic progenitors during ageing

**Figure 1:**
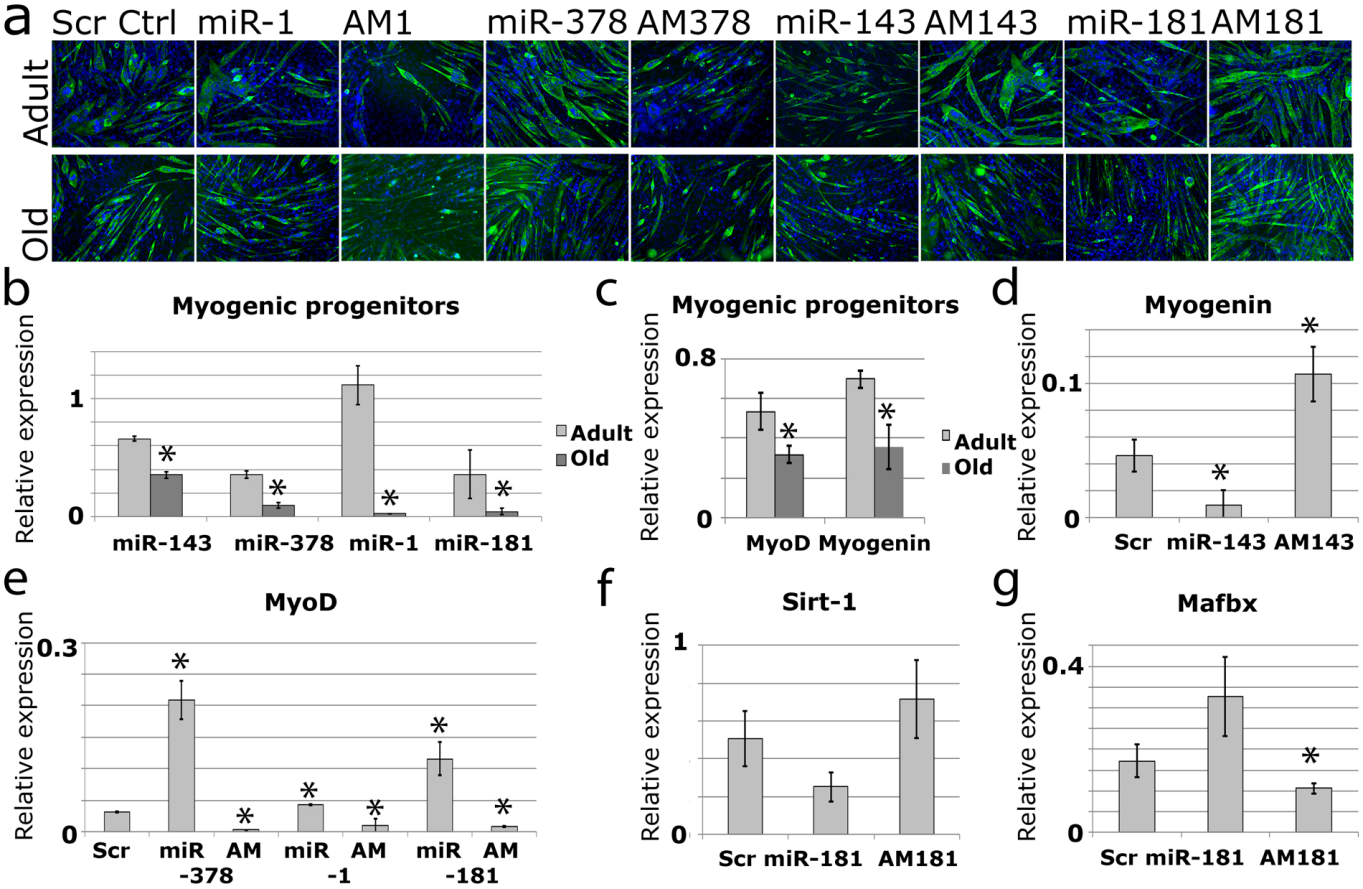
The role of selected miRNAs in regulating adult myogenesis. (**a**) MF20 immunostaining showing miRNA regulation of myogenic differentiation of primary myoblasts from adult or old mice. Green – MF20, blue – DAPI. (**b**–**g**) qPCR showing changes in miRNA and gene expression in murine myogenic progenitors during ageing or following microRNA overexpression (mimic, miR) or inhibition (antimiR, AM). Expression relative to Rnu-6 (miRNA) or β-2 microblobulin (mRNA) is shown, *p < 0.05 as compared to Adult/Adult scr; Error bars show SEM; n = 3. Adult – 6 months old, Old – 24 months old, Scr – antimiR scrambled.

In order to create a model of the effects of selected miRNAs on myogenesis, we first overexpressed and inhibited these miRNAs in undifferentiated C2C12 cells. Overexpressing miR-1, miR-181, or miR-378 improved the efficiency of myogenesis, whereas increased levels of miR-143 decreased myogenesis efficiency as compared to scrambled-transfected controls (Fig. 1a). Conversely, inhibition of the miRNAs led to the opposite effect than their overexpression. In addition, all four miRNAs and gene expression of MyoD and Myogenin were down-regulated in myogenic progenitor cells during ageing (Fig. 1b,c). Experiments were also carried out to validate the model output (Fig. 1d,f), which are described below.

### Model 1: miR-1 promotes the switch from quiescence to differentiation

**Figure 2:**
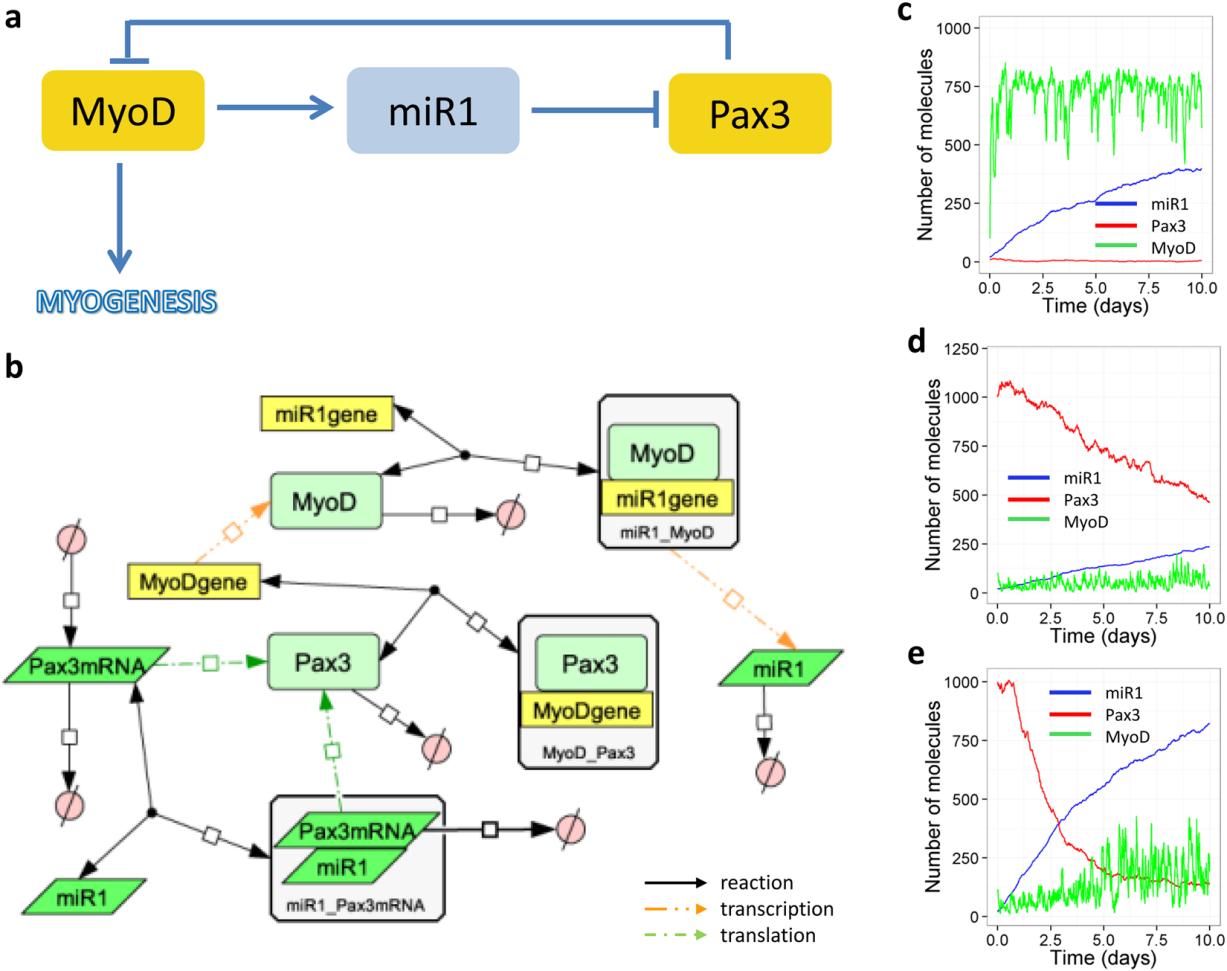
A model of miR-1 in muscle. (**a**) Diagram showing the negative feedback loop. (**b**) Network diagram of full model. (**c**–**e**) Model output. (**c**) Low Pax3 (default parameters). (**d**) High Pax3 (initially Pax3 = 1000, Pax3mRNA = 400, *k*_*synPax3*_ = 6.0e-5 s^−1^, *k*_*synPax3mRNA*_ = 0.08 molecules s^−1^). (**e**) High Pax3 and increased production of miR-1 (parameters as in D except *k*_*synmiR1*_ = 0.001). Default values for initial amounts and parameters are shown in Supplementary Tables S1 and S2 respectively. A larger version of Fig. 2b is shown in Supplementary Figure S15.

miR-1 expression is downregulated in myogenic progenitors during ageing (Fig. 1a,b). We constructed a computational model in which we assumed that MyoD upregulates miR-1 expression^14^ and that miR-1 enhances the degradation of Pax3 mRNA^13^. In addition, as Pax3 expression was found to be negatively correlated with MyoD^13^ we assumed that Pax3 inhibits transcription of MyoD resulting in a negative feedback loop (Fig. 2a,b). Experimental data using a glioma cell line which does not express miR-1 but expresses high levels of Pax3 showed that transfecting cells with miR-1 led to reduced levels of both Pax3 transcript and protein at 48 h^13^. We validated our model against this data by running simulations for five hours (virtual time) in the total absence of miR-1 and compared this to our results in which MyoD induces miR-1 expression. In order to fit our model to this data, it was necessary to assume that miR-1 enhances degradation of Pax3 transcript but does not inhibit Pax3 translation. Therefore, we needed to include a reaction in which Pax3 mRNA bound by miR-1 could be translated into protein. The model output shows that the presence of miR-1 leads to about a 30–50% reduction in Pax3 transcript and protein at 48 h, which is in good agreement with the experimental data^13^ (Supplementary Figure S1).

After validating the model, we simulated the effect of high and low levels of Pax3 on myogenesis over a 10-day period. As expected, low levels of Pax3 are associated with high levels of MyoD and miR-1 ensuring that Pax3 levels remain low and myogenesis can occur (Fig. 2c). Conversely, high Pax3 levels are associated with low levels of MyoD and miR-1 enabling Pax3 expression to remain high (Fig. 2d). In addition, we examined the effect of miR-1 on the balance between proliferation, quiescence and differentiation. It should be noted that the model also includes a reaction in which miR-1 is produced at a very low rate independently of MyoD. If the parameter for this reaction is increased, miR-1 expression gradually increases over time, despite the low levels of MyoD, and so Pax3 levels decrease, leading to release of the inhibition of MyoD which eventually increases. These interactions lead to a further increase in miR-1 levels and further inhibition of Pax3 expression (Fig. 2e). This model suggests that a threshold level of miR-1 may play a role in the initial step of myogenesis: a switch from a quiescent to a differentiating satellite cell. Our experimental data supported both the role of miR-1 on myogenic differentiation of myogenic progenitors from adult and old mice, and the positive regulation of MyoD expression by miR-1 (Fig. 1).

### Model 2: a threshold level of miR-181 enhances myogenesis but may also increase muscle atrophy or inhibit muscle hypertrophy

**Figure 3:**
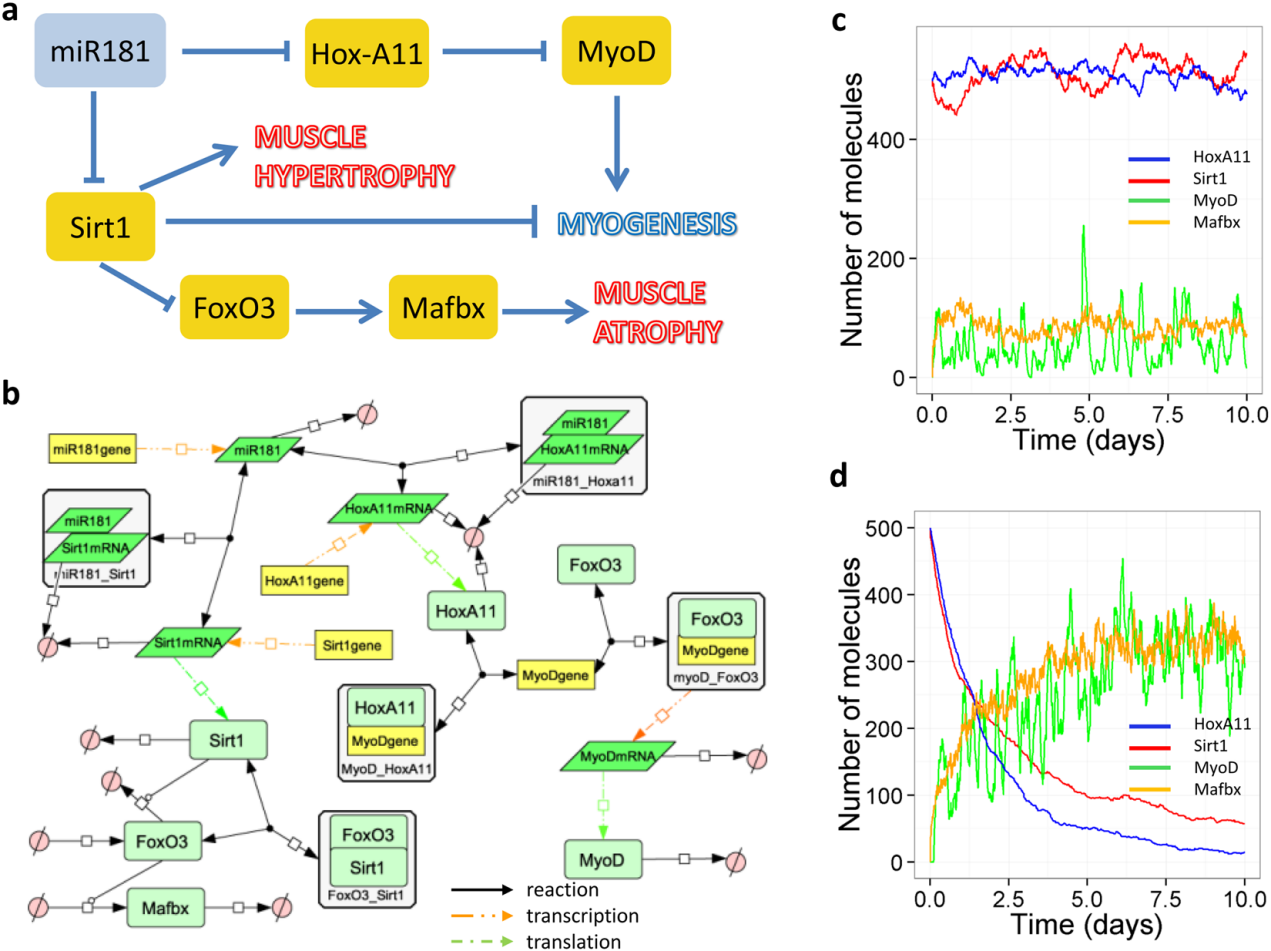
A model of miR-181 in muscle. (**a**) Diagram showing how miR-181 positively regulates myogenesis. (**b**) Network diagram of full model. (**c**,**d**) Model output of miR-181 model showing Hox-A11, Sirt-1, Mafbx and MyoD protein levels. (**c**) Low miR-181 (default parameters). (**d**) High miR-181 (initially miR-181 = 1000, *k*_*synmiR181*_ = 6e-4 s^−1^). (**c**,**d**) Show output from one individual stochastic simulation. Default values for initial amounts and parameters are shown in Supplementary Tables S3 and S4 respectively. A larger version of Fig. 3b is shown in Supplementary Figure S16.

The expression of miR-181 is downregulated in myogenic progenitors during ageing (Fig. 1a,b). We constructed a model of the miR-181-associated network to demonstrate its positive regulation of myogenesis by activating MyoD and inhibiting Sirt-1 expression (Fig. 3a). We assumed that Hox-A11 inhibits MyoD expression^27^ by binding to its promoter to inhibit MyoD transcription, and that miR-181 inhibits Hox-A11 expression at the protein levels but did not enhance degradation of Hox-A11 mRNA^12^ (Fig. 3b). In addition, we assumed that miR-181 inhibits translation of Sirt-1 and targets Sirt-1 mRNA for degradation but does not enhance its degradation rate^20^. We also included inhibition of FoxO3 by Sirt-1^23^ in the model and assumed that FoxO3 upregulates the levels of Mafbx^28^, which leads to muscle atrophy due to increased protein degradation. We carried out simulations of the model with either low or high levels of miR-181 (Fig. 3c,d). Low levels of miR-181 lead to high levels of both Hox-A11 and Sirt-1, and low levels of MyoD (Fig. 3c). The converse is true when miR-181 expression is high (Fig. 3d). Previous studies have shown that miR-181 affects protein, but not mRNA, levels of Sirt-1 and Hox-A11^12,20^. Therefore, we validated our model to check whether it agreed with the experimental data (Supplementary Figure S2). As in the published data, our model showed that mRNA levels of Sirt-1 or Hox-A11 are unaffected by changing the level of miR-181 but protein levels significantly declined when miR-181 levels increased. According to our model, as Sirt-1 protein levels decline in the presence of high levels of miR-181, FoxO3 is released from the inhibitory effects of Sirt-1 and is able to translocate to the nucleus and transcribe genes such as Mafbx. Mafbx is an E3 ubiquitin ligase that enhances the degradation of muscle-specific proteins leading to muscle atrophy, and so miR-181 may be detrimental in regulating muscle atrophy (Fig. 3c,d). Our experimental data supported the role of miR-181 on myogenic differentiation of myogenic progenitors from adult and old mice through MyoD (Fig. 1e) and also the role of miR-181 on myotube atrophy/hypertrophy through regulating Sirt-1 and Mafbx expression (Fig. 1f).

### Model 3: upregulation of miR-378 by MyoD releases the inhibitory effect of Musculin on MyoD activity

**Figure 4:**
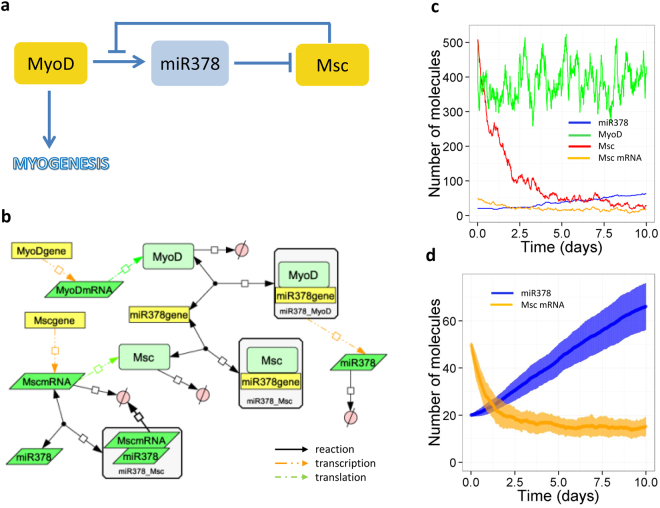
A model of miR-378 in muscle. (**a**) Diagram showing positive auto-regulation of MyoD activity. (**b**) Network diagram of full model. (**c**,**d**) Model output showing inverse relationship between MyoD activity (indicated by levels of miR-378) and Msc levels. (**c**) One individual simulation. (**d**) Mean (bold curves) and standard deviation (shaded area) from 100 simulations. Default values for initial amounts and parameters are shown in Supplementary Tables S5 and S6 respectively. A larger version of Fig. 4b is shown in Supplementary Figure S17.

The expression of miR-378 is downregulated in myogenic progenitors during ageing (Fig. 1). In the miR-378-associated model, we assumed that MyoD initiates transcription of miR-378^11^, that miR-378 targets Msc mRNA for degradation^11^, and that Msc binds to the miR-378 gene to prevent MyoD binding^29^, i.e. it inhibits MyoD transcriptional activity (Fig. 4a,b). The model simulation output shows that as miR-378 level increases, a simultaneous decrease in Msc levels occurs, therefore the levels of miR-378 and Msc are approximately anti-correlated (Fig. 4c,d), consistent with experimental data^11^. We also examined the effect of increasing or inhibiting miR-378 levels by varying the parameters which control miR-378 synthesis. Increasing the rate of binding of the miR-378 gene by Msc led to lower levels of miR-378, as expected, and led to an increase in Msc mRNA levels (Supplementary Figure S3). However, stochastic simulation indicates that there is large variability in levels of miR-378 and Msc mRNA with the same parameter value, although the average behaviour is similar to the deterministic result (Supplementary Figure S3). Increasing/inhibiting MyoD activity was next simulated by varying the rate of binding of MyoD to the miR-378 gene. As MyoD activity increased, miR-378 levels increased, as expected, and Msc mRNA levels decreased (the opposite result to varying the rate of binding of Msc to the miR-378 gene) as shown (Supplementary Figure S3). Therefore, miR-378 may act in the differentiation process to maintain MyoD activity. Our experimental data supported the role of miR-378 on myogenic differentiation of myogenic progenitors from adult and old mice through regulation of MyoD levels (Fig. 1e).

### Model 4: miR-143 is inhibited by prolonged IL-6 signalling resulting in higher levels of myogenin

**Figure 5:**
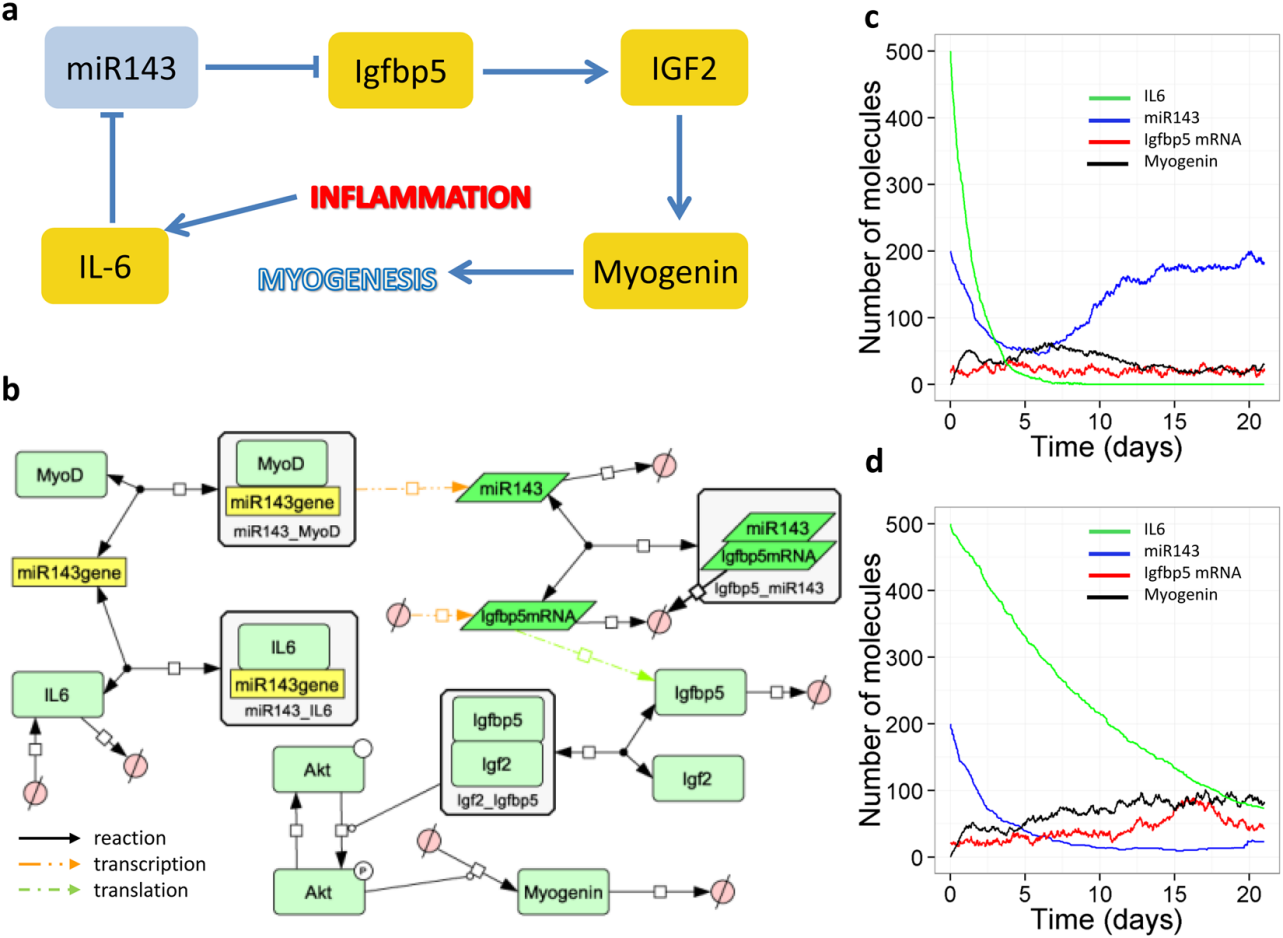
A model of miR-143 in muscle. (**a**) Diagram showing key components and interactions. (**b**) Network of full model. (**c**,**d**) Model output. (**c**) Transient IL-6 response (default parameters). (**d**) Slower decline of IL-6 leads to prolonged signalling (*k*_*degIL6*_ = 1e-6 s^−1^). Default values for initial amounts and parameters are shown in Supplementary Tables S7 and S8 respectively. A larger version of Fig. 5b is shown in Supplementary Figure S18.

The expression of miR-143 is downregulated in myogenic progenitors during ageing (Fig. 1). We modelled the effects of miR-143 in myogenesis by assuming that miR-143 expression is down-regulated by IL-6^25^ and that miR-143 inhibits Igfbp5 expression by both enhancing degradation and inhibiting translation of Igfbp5 mRNA^25^. Igfbp5 binds to IGF2 and enhances IGF2 binding to the IGF1-R receptor^30^. This activates Akt signalling, by increasing phosphorylation of Akt, leading to upregulation of myogenin expression^30^. We modelled IL-6-mediated inhibition of miR-143 by assuming that IL-6 binds to the miR-143 gene to inhibit its transcription. This is a simplifying assumption; however the model could be extended when details of the mechanism are better known. A simplified diagram and a network of the full model are shown in Fig. 5a,b. We assumed that IL-6 levels increase in response to injury and ran simulations by assuming that initially IL-6 levels are high and then decline due to negative feedback loops in the IL-6 signalling pathway. In this scenario, miR-143 levels rapidly decline in response to IL-6 but by 21 days (as shown experimentally following muscle injury^25^), miR-143 levels return to normal (Fig. 5c). In old tissues, a chronic activation of inflammatory pathways may exist and we modelled this by assuming that IL-6 levels show a much slower decline rate after activation, due to dysregulation of feedback loops during ageing. In this situation, miR-143 levels decline and remain low even at 21 days following injury (Fig. 5d). This agrees well with our experimental data and supports the hypothesis that down-regulation of miR-143 in ageing is a compensatory mechanism to help alleviate the decline in muscle regeneration with age^25^. To examine the effect of IL-6 on miR-143 levels, we varied its synthesis rate with both transient and sustained IL-6 signalling (Supplementary Figure S4). Due to the inhibitory effect of IL-6 on miR-143 transcription, miR-143 levels only increase when IL-6 levels are low, even at higher miR-143 synthesis rates. Therefore, it can only increase when IL-6 signalling is transient as shown in Supplementary Figure 4. Our experimental data supported the role of miR-143 on myogenic differentiation of myogenic progenitors from adult and old mice through regulation of myogenin levels (Fig. 1d).

### Model 5: Integrated model

**Figure 6:**
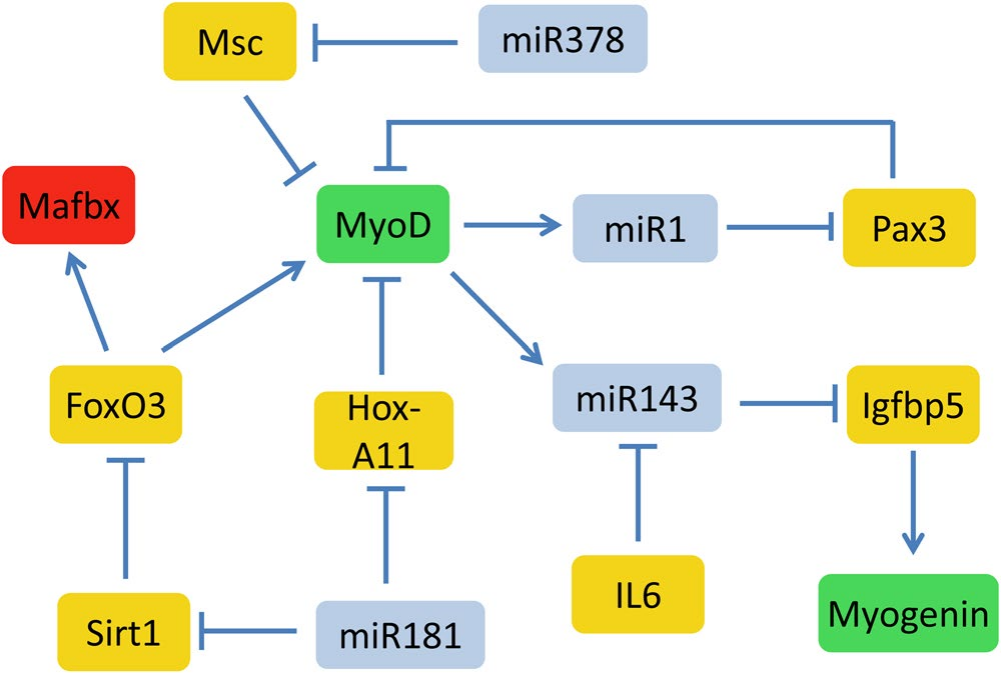
Simplified network diagram of integrated model. Key: blue boxes: miRNAs, green boxes: proteins that activate myogenesis; red boxes: outputs representing atrophy; mustard boxes: other regulatory proteins.

We combined all four models to form an integrated model. As miR-1 and miR-143 are MyoD targets, we also added inhibition of miR-1 and miR-143 transcription by Msc. A simplified diagram of the network is shown in Fig. 6. When we simulated the integrated model with all the default parameters from the individual models, the simulation output showed that even when Pax3 levels are low, MyoD is also low (Supplementary Figure 5). This is due to the high levels of Hox-A11 which inhibits MyoD expression (Supplementary Figure 5). If we assume that miR-181 levels are high, Hox-A11 levels decrease resulting in a recovery of MyoD levels and this in turn leads to an increase in miR-1 expression (Supplementary Figure 6). In addition, miR-378 levels slightly increase, resulting in lower levels of Msc, and miR-143 levels also increase after IL-6 levels have declined (compare Supplementary Figures 5 and 6). Since MyoD is also inhibited by Msc, we modelled the effect of high levels of miR-378 when miR-181 is low (Supplementary Figure 7). With lower levels of Msc, MyoD levels increase (as can be seen by the resulting higher levels of miR-1) but the effect is modest as Hox-A11 levels are high. If miR-181 and miR-378 levels are both high, then there is no significant effect on MyoD levels but miR-1 is further increased due to less inhibition of MyoD activity by Msc (Supplementary Figure 8). We then simulated the model under conditions of high Pax3 with miR-181 and miR-378 set at low levels (Supplementary Figure 9) and as expected MyoD levels are very low. Increasing both miR-181 and miR-378 under these conditions led to a modest recovery in MyoD levels and increased activity (as seen by an increase in miR-1 and miR-378) and at late timepoints miR-143 starts to increase (Supplementary Figure 10). Increasing miR-1 levels and its synthesis rate at the start of the simulation results in a faster decline in Pax3, and MyoD levels start to recover earlier although they do not seem to reach higher levels (Supplementary Figure 11).

We varied the transcription rate of miR-143 with both transient and sustained IL-6 signalling and the effect was similar to that obtained in the individual model, except that a higher rate of miR-143 transcription is needed due to the additional inhibition of MyoD in the integrated model by both Hox-A11 and Msc (Supplementary Figure 12). However, increasing both miR-181 and miR-378 resulted in higher miR-143 levels and the results of varying *k*_*synmiR143*_ in the integrated model is then very similar to the individual model (Supplementary Figure 13). Finally, we varied the rate of miR-378 transcription and the model output showed that decreasing miR-378 transcription resulted in higher levels of Msc protein (with a slight increase in mRNA) but no effect on MyoD levels (Supplementary Figure 14). There was also lower levels of miR-1 but no effect on miR-143 or miR-181 levels (Supplementary Figure 14). Conversely increasing miR-378 transcription led to lower levels of Msc protein, no effect on MyoD levels and higher levels of miR-1. In addition, miR-143 levels showed a slight recovery after 9 days but miR-181 levels were still unaffected (Supplementary Figure 14).

## Discussion

We have developed a set of models to illustrate how computational modelling can be used as an additional tool for investigating the behaviour of miRNA:target interactions in muscle homeostasis, specifically in adult myogenesis. To build a model that starts to decipher ageing-associated changes in muscle regeneration, we selected four different miRNAs known to be important in muscle homeostasis in adulthood and/or ageing and first built individual models of each one, based on current experimental data relevant to muscle regeneration. At this stage, we kept the models as simple as possible but as they have been developed using the Systems Biology Markup Language (SBML) modelling standard^31^, they are very amenable to modification and can be extended to included more interactions as required, for example as new data emerge from experimental studies. Other computational approaches have recently been developed which may provide further information for the models. In particular, methods for predicting miRNAs associated with disease using miRNA-disease heterogeneous networks have been developed, e.g.^32,33^, and are the focus of three recent reviews^34–36^.

Computational modelling has previously been extensively used in the field of cancer (reviewed in^37^), and also established as a tool in modelling congenital heart disease (reviewed in^38^). Despite the success of modelling in these fields, there is still a paucity of computational models in the study of human diseases. In particular, to our knowledge, there is currently a lack of computational models of the underlying molecular mechanisms leading to muscle dysfunction in ageing and disease. Our method has the potential to be used to help increase our understanding of other diseases related to muscle such as Duchenne Muscular Dystrophy (DMD) and myositis. DMD is a severe type of muscular dystrophy for which there is currently no cure. It is caused by a mutation in the dystrophin gene, leading to dysregulation in calcium and other signalling pathways, mitochondrial dysfunction and necrosis of muscle fibres. However, the underlying pathways are complex and not fully understood, suggesting the need for computational modelling. Considering the limited success of current therapies undergoing clinical trials, additional computational approaches could possibly be used to enhance the success of exon skipping or to propose other therapies. Myositis is charactersied by the infiltration of immune cells into the muscle leading to chronic inflammation, however, the mechanisms by which damage to muscle occurs is poorly understood. Particularly, the mechanism by which changes in the immune cells infiltrating the muscle and changes within the muscle tissue itself contribute to the onset of myositis are not understood. We suggest that computational modelling could be useful in simulating the effects of an increase in pro-inflammatory cytokines on the function of muscle cells.

The miR-1 model illustrates how MyoD initiates a positive feedback mechanism to ensure that its expression is switched on and remains on during mygonesis. mir-1 is central to this switch as it is upregulated by MyoD and then targets Pax3, an inhibitor of MyoD. This model could be extended in various ways. First, it would be interesting to include dimerisation of MyoD, since this is required for its transcriptional activity. This would affect the concentration of MyoD required for its activity and would help to prevent inappropiate upregulation of genes when MyoD is at low concentrations. It has also been shown that miR-1 targets Hdac4^39^, a histone deacetylase which represses Mef2C, an essential myogenic transcription factor. This would provide an additional pathway by which miR-1 promotes myogenesis. Furthermore, a study has shown that miR-1 targets Hsp70 to antagonise the inhibitory effect of Hsp70 on Akt phosphorylation^40^. Phosphorylated Akt, inactivates Foxo3 which in turn inhibits two E3 ubiquitin ligases, Murf1 and Atrogin-1, both of which are implicated in muscle atrophy due to increased protein degradation^28^.

The miR-181 model suggests that above a threshold level, miR-181 promotes myogenesis, however it may also lead to either muscle atrophy or inhibition of hypertrophy. This is due to the inclusion of two targets: Hox-A11 and Sirt-1, which both inhibit myogenesis via different pathways. In addition, Sirt-1 inhibits muscle atrophy and promotes hypertrophy. Therefore, the effect of miR-181 via Sirt-1 is complex and requires further investigation into what determines the different outcomes due to Sirt-1. External conditions such as mechanical signals and nutrient supply regulate Sirt-1 activity and so the effect of Sirt-1 may depend on the energy status of the cell. (reviewed in^41^). Sirt-1 activity is also dysregulated in ageing leading to lower expression of antioxidants and increased oxidative stress^42^. Moreover, Sirt-1 has been shown to positively regulate metabolic state of satellite cells and autophagy, which is also associated with FoxO3 activity^43^. Therefore, including more detail of the pathways involving Sirt-1 would be important in the context of modelling age-related changes in muscle regeneration.

Similarly to miR-1, we modelled the role of miR-378 in MyoD positive auto-regulation. Since many miRNAs are thought to have fine-tuning effects on gene expression, it is not surprising that there are several miRNAs working in similar pathways. Whereas miR-1 inhibits Pax3 expression which down-regulates MyoD, miR-378 inhibits Musculin, a transcription factor that binds to the same target genes as MyoD, thereby inhibiting MyoD activity. Therefore, increased expression of both miR-1 and miR-378 would result in increased expression and higher activity of MyoD. Our model of miR-378 is currently simple but could be expanded to include its role in metabolism. The gene for miR-378 is in the first intron of Pgc-1β and so is expressed more in metabolically active tissue and it has been shown to activate the pyruvate-PEP futile cycle in skeletal muscle of mice whereby it enhances lipolysis in adipose tissue and ameliorates obesity^44^. A further study by the same group showed that miR-378 targets Igfr1 in mice leading to defective muscle regeneration^45^.

In the first three models, the miRNAs had mainly postive effects on muscle regeneration, whereas miR-143 has been shown to have detrimental effects as it targets components of the insulin signalling pathway leading to inhibition of myogenesis. Interestingly, miR-143 is downregulated in ageing which may be due to chronic inflammation. Our model was able to simulate the effects on miR-143 inhibition on myogenesis and showed that low miR-143 levels led to higher levels of Igfbp5 and an increase in myogenin (the model readout for myogenesis) in agreement with our experimental data (Fig. 1d) and a previous study^25^. The same study showed that miR-143:Igfbp5 interactions regulate myoblast senescence. The model could be developed further to study this by adding components of the senescent pathway such as p16 and Rb. Finally adding in further detail of the IL-6 pathway may help to unravel its complex signalling pathways in muscle since it has been shown to have mainly beneficial but also detrimental effects^46^.

We illustrated the approach with miR-1, miR-181, miR-378 and miR-143 but intend to develop further models of other miRNAs. For example, miR-23a and miR-182 regulate expression of atrophy genes^47,48^, miR-128 downregulates genes involved in the insulin/IGF1 signalling pathway in skeletal muscle^49^, and miR-206, a miRNA in the same family as miR-1 also targets Pax3^13^. For a comprehensive discussion of the role of miRNAs in skeletal muscle we refer the reader to recent reviews^10,50^. Since miRNAs are involved in many different gene regulatory circuits and affect multiple signalling pathways, it will be important to investigate the cross-talk between miRNAs, their regulators and their targets. Computational modelling can contribute to this by combining models of individual miRNAs into an integrated model and then simulate the effect of perturbing one or more components on the overall behaviour of the system. We illustrated this approach by combining each of the models described in this paper into an integrated model. The output from this model showed that crosstalk between the pathways affected levels of miRNAs themselves, especially those that are regulated by MyoD, due to different pathways having an inhibitory effect on MyoD activity. In addition, we examined the effects of overexpressing each miRNA individually and also in combination and showed that miR-1, miR-181 and miR-378 all have positive effects on myogenesis, whereas overexpression of miR-143 leads to detrimental effects due to lower levels of myogenin. This is consistent with our experimental data (Fig. 1). It has been suggested that down-regulation of miR-143 with age may be a compensatory effect and our model supported this idea with miR-143 levels declining in response to persistent IL-6 signalling. It would be interesting to extend our model to include other mechanims of ageing such as oxidative damage, redox regulation of miRNA genes and dysregulation of transcriptional regulation and to simulate the model over longer timescales to represent an ageing organism. It would also be important to study the effects of ageing on muscle in relation to physical activity, since a decline in activity is common in ageing and exarcerbates the loss of muscle mass and function^51^.

Although we have modelled complex processes, such as ageing during which multiple mechanistic and compensatory changes within tissues and surrounding environemnt occur, we kept the models as simple as possible but included enough detail so that they are supported by the experimental data in a meaningful way. It is possible to improve the fit of the models to the data by making them more complex. This, however, may then have the disadvantage of not being useful for interpreting the underlying dynamics of the system (a problem commonly caused by over-fitting) or for making predictions. It should be pointed out that we do not claim that we have developed the “best models” as modelling is a dynamic process and future research may produce new data which is inconsistent with our models. In this case the model output may provide clues as to what is missing or incorrect in the models so that modifications can be made. SBML models are very amenable to this process and examples of adapting models for new data has been illustrated in the literature.

Finally, there are major advantages of using computational modelling from both financial and ethical perspectives. For example, computer simulation can be used to investigate the effects of single and multiple targets before *in vivo* studies, so that only targets that have been predicted by the model to be beneficial need to tested. The models can also take into account redundancy as has been observed for miR-1 and miR-206 in which single knock-outs in mice have hardly any phenotype^13,52^, highlighting the possible need for a multiple target approach. Finding the best combination of multiple targets may be infeasible and unethical in an experimental setting but well-suited to a computational approach.

## Methods

### Model construction

All models were constructed using SBML shorthand^53^ and a Python tool to convert the code into SBML^31^. Simulations were performed in COPASI 4.18^54^ using either the deterministic LSODA algorithm^55^ or the stochastic direct method which is based on the Gillespie algorithm^56^. The LSODA algorithm solves stiff and non-stiff systems of ordinary differential equations by automatic selection^55^. We used the COPASI default parameters for this solver (relative tolerance = 1e-6; absolute tolerance = 1e-13; maximum internal step size = 10000). The stochastic Gillespie direct method simulates every reaction, updating the number of molecules of each species after a reaction occurs. At each time-step a random number is generated to determine the next reaction to occur and the time interval to that reaction. The probability of any particular reaction occurring is proportional to its associated parameter value multiplied by the number of substrate molecules. Each stochastic simulation produces a different trajectory. Therefore multiple stochastic simulations were also carried out on a cluster using software developed at Newcastle University^57^ which also used the Gillespie algorithm. This software is encoded in the C programming language and is available on request. Simulation data were analysed and plotted in R using ggplot2^58^. Network diagrams of the models were created in CellDesigner^59^ which used the Systems Biology Graphical Notation^60^.

### Isolation of primary myoblasts from mouse skeletal muscle

Primary myoblasts from adult (6 months old) and old (24 months old) mice were prepared from EDL muscles via collagenase and dispase digestion as described by us previously^61^. This allowed for isolation of a pure population of primary myoblasts retaining the expression patterns of selected miRNAs.

### Cell culture

Mouse primary myoblasts were maintained in DMEM media supplemented with 20% fetal bovine serum, 10% horse serum and 1% penicillin/streptomycin. To induce myogenic differentiation 90% confluent cells were cultured in DMEM supplemented with 5% horse serum and 1% penicillin/streptomycin (DM). Myoblast differentiation was assessed 10 days following the switch to DM by immunostaining for myosin heavy chain using the MF20 antibody^52^. Images were taken using Zeiss fluorescent microscope and analysed using ImageJ as in^25^.

### Transfections

Myoblasts were transfected with 100 nM miRNA mimic or antimiR (Qiagen) or scrambled antagomiR not predicted to bind to any know miRNA^13^, as indicated in figures, using Lipofectamine 2000^TM^ ^52^. AntimiR-scrambled-transfected cell served as controls. This provided a transfection efficiency of 40–70%, depending on the molecule transfected as described previously^52^.

### Real-Time PCR

RNA isolation and quantitative real time PCR were performed using standard methods as described previously^62^. cDNA synthesis (mRNA) was performed using 500ng RNA and SuperScript II according to the manufacturer’s protocol (Invitrogen). cDNA synthesis (miRNA) was performed using 100ng RNA and miRscript RT kit II according to the manufacturer’s protocol (Qiagen). qPCR analysis was performed using miRScript SybrGreen Mastermix or sso-Advanced SybrGreen Mastermix in a 20 µl reaction (Qiagen or Biorad, respectively). Expression relative to β-2 microblobulin and/or 18S (mRNA) or Rnu-6 and/or Snord-61 (miRNA) was calculated using delta delta Ct method.

### Mice

The study was performed using male wild type C57Bl/6 mice (adult: 6 months old; old – 24 months old). Mice were obtained from Charles River (Margate). All mice were maintained under specific-pathogen free conditions and fed ad libitum a standard chow and maintained under barrier on a 12-h light-dark cycle. For tissue collection, mice were culled by cervical dislocation. The tissues were immediately excised, frozen and stored at −80 °C. Experiments were performed in accordance with UK Home Office guidelines under the UK Animals (Scientific Procedures) Act 1986 and received ethical approval from the University of Liverpool Animal Welfare and Ethical Review Body (AWERB). Primary myoblasts from adult (7 months old) and old (24 months old) mice were prepared from EDL muscles following single fibre isolation as previously described^63,64^. These myoblasts retained the expression patterns of miRNAs.

### Data Availability

The SBML code for each of the four individual models and the integrated model was deposited in the public repository, Biomodels^65^ and assigned the identifiers MODEL1704110000-MODEL1704110004.

## Electronic supplementary material

Supplementary Information
