# Author Correction: Using computer simulation models to investigate the most promising microRNAs to improve muscle regeneration during ageing

**DOI:** 10.1038/s41598-023-31165-y

**Published:** 2023-03-16

**Authors:** Carole J. Proctor, Katarzyna Goljanek-Whysall

**Affiliations:** 1 MRC/Arthritis Research UK Centre for Musculoskeletal Ageing (CIMA), Institute of Cellular Medicine and Newcastle University Institute for Ageing, Newcastle University, Newcastle Upon Tyne, UK; 2 MRC/Arthritis Research UK Centre for Musculoskeletal Ageing (CIMA), Department of Musculoskeletal Biology, Institute of Ageing and Chronic Disease, University of Liverpool, Liverpool, UK

Correction to: *Scientific Reports* https://doi.org/10.1038/s41598-017-12538-6, published online 26 September 2017

The original version of this Article contains an error in Figure 1 panel A, where the image representing Adult AM143 is incorrect.

The correct Figure 1 and accompanying legend appear below.

**Figure 1:**
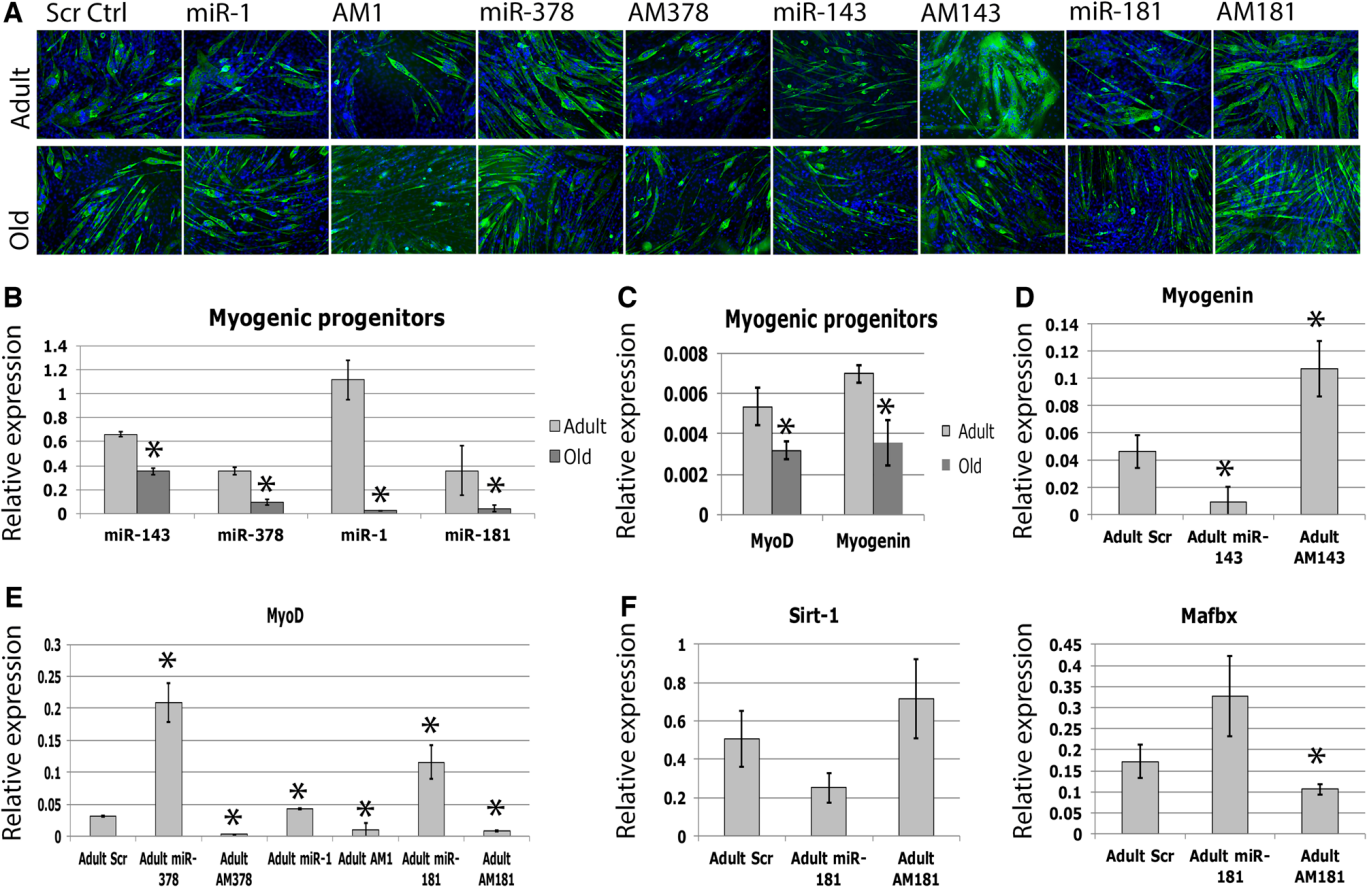
The role of selected miRNAs in regulating adult myogenesis. (**a**) MF20 immunostaining showing miRNA regulation of myogenic differentiation of primary myoblasts from adult or old mice. Green – MF20, blue – DAPI. (**b–g**) qPCR showing changes in miRNA and gene expression in murine myogenic progenitors during ageing or following microRNA overexpression (mimic, miR) or inhibition (antimiR, AM). Expression relative to Rnu-6 (miRNA) or β-2 microblobulin (mRNA) is shown, *p < 0.05 as compared to Adult/Adult scr; Error bars show SEM; n = 3. Adult – 6 months old, Old – 24 months old, Scr – antimiR scrambled.

