# Supplementary material for: Using computer simulation models to investigate the most promising microRNAs to improve muscle regeneration during ageing
Source: Sci Rep. 2017 Sep 26;7:12314. doi: 10.1038/s41598-017-12538-6 (PMC5614911; doi:10.1038/s41598-017-12538-6)
Supplement: Supplementary file 1 — Supplementary Information [file 41598_2017_12538_MOESM1_ESM.pdf]

Supplementary Information

Using computer simulation models to investigate the most promising microRNAs to improve muscle regeneration during ageing

Carole J Proctor[a*], Katarzyna Goljanek-Whysall[b]

[a]MRC/Arthritis Research UK Centre for Musculoskeletal Ageing (CIMA), Institute of Cellular Medicine and Newcastle University Institute for Ageing, Newcastle University, Newcastle upon Tyne UK. [b] MRC/Arthritis Research UK Centre for Musculoskeletal Ageing (CIMA), Department of Musculoskeletal Biology, Institute of Ageing and Chronic Disease, University of Liverpool, Liverpool, UK

Figure S1. Validating the miR-1 model against data for Rugli cells. The Rugli cell line does not express miR-1 but expresses high levels of Pax3[1]. Parameters were set for high Pax3 (Pax3=1000, Pax3mRNA=400, $k_{synPax3}$=6e-5 mol s^{-1}, $k_{synPax3mRNA}$=0.08 s^{-1}). (**a**) No miR-1 (miR1=0, $k_{synmiR1}$=0 s^{-1}, $k_{synmiR1MyoD}$=0 s^{-1}); (**b**) Addition of miR1 (miR1=1000, $k_{synmiR1}$=1e-4 s^{-1}, $k_{synmiR1MyoD}$=5e-4 s^{-1}). Two individual stochastic simulations are shown.

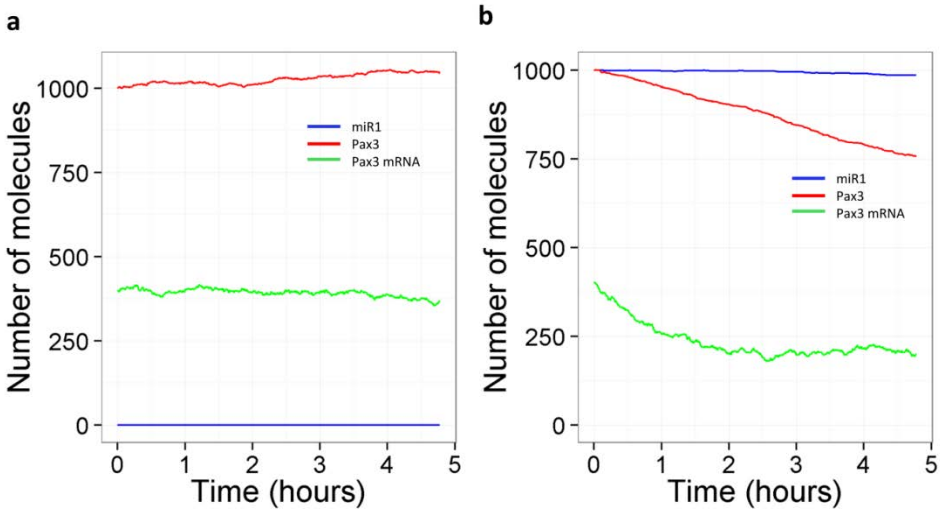

Figure S2. Validating the miR-181 model against experimental data. Experimental data shows that miR-181 affects HoxA11 and Sirt1 protein levels but not mRNA levels[2,3]. The model was simulated over a ten day time period with low levels of miR-181 (miR181=10, $k_{synmiR181}$=6e-6 s^{-1}) or high levels of miR-181 (miR181=1000, $k_{synmiR181}$=6e-4 s^{-1}) and the mean levels of Sirt1 and HoxA11 protein and mRNA from 1000 time-points were calculated. Stochastic simulation was used and the mean of 10 runs are shown.

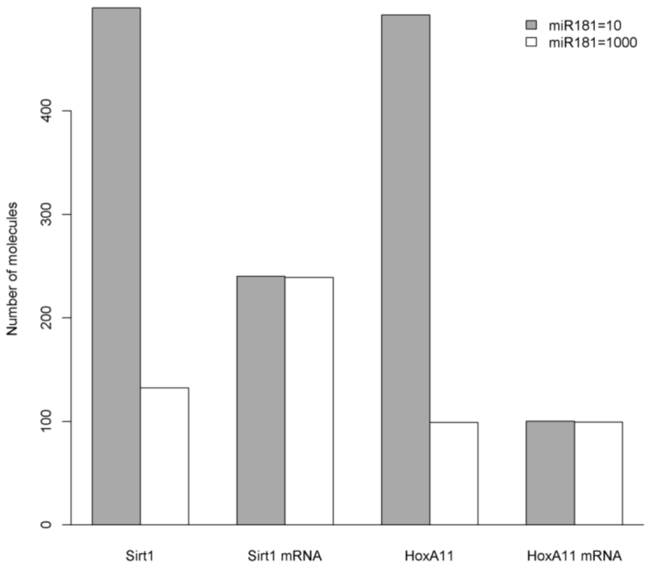

Figure S3. Simulating the effect of increasing/inhibiting miR-378 in the miR-378 model. (**a**) Deterministic model with $k_{binmiR378geneMsc}$ varying from 1e-6 to 1e-4 $mol^{-1}s^{-1}$ using logarithmic scale (default value =1e-5 $mol^{-1}s^{-1}$) indicated by increasing thickness of the lines in the plot. (**b-c**) stochastic model with $k_{binmiR378geneMsc}$=1e-6 or 1e-4 $mol^{-1}s^{-1}$ respectively, output from 100 simulation, thick lines show mean values. (**d**) Deterministic model with $k_{binmiR378geneMyoD}$ varying from 1e-6 to 1e-4 using logarithmic scale (default value =1e-5 $mol^{-1}s^{-1}$) indicated by increasing thickness of the lines in the plot. **b-c** Stochastic model with $k_{binmiR378geneMyoD}$=1e-6 or 1e-4 $mol^{-1}s^{-1}$ respectively, output from 100 simulations, thick lines show mean values. Range of values of $k_{binmiR378geneMsc}$ and $k_{binmiR378geneMyoD}$ are indicated at side of deterministic plots in (**a**) and (**d**) respectively. (mol = number of molecules).

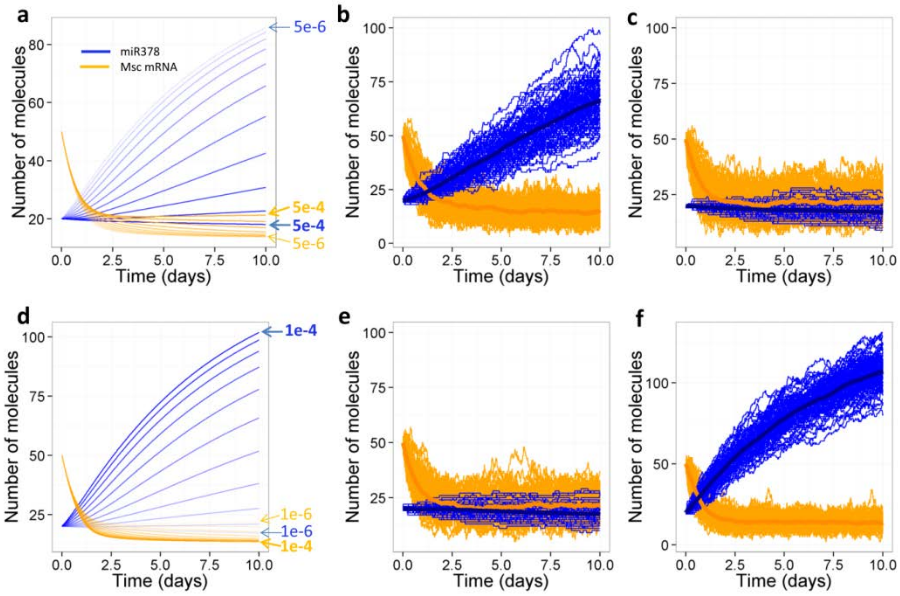

Figure S4. The effect of varying miR-143 synthesis rate when IL-6 signalling is transient or sustained in the miR-143 model. (**a-c**) Transient IL-6 signalling (k_{degIL6}=8e-6 s^{-1}). (**d-f**) Sustained IL-6 signalling (k_{degIL6}=1e-6 s^{-1}). (**a**,**d**) $k_{synmiR143}$=5e-5 s^{-1}. (**b**,**e**) $k_{synmiR143}$=5e-4 s^{-1} (default value). (**c**,**f**) $k_{synmiR143}$=5e-3 s^{-1}. Ten individual stochastic simulations are shown.

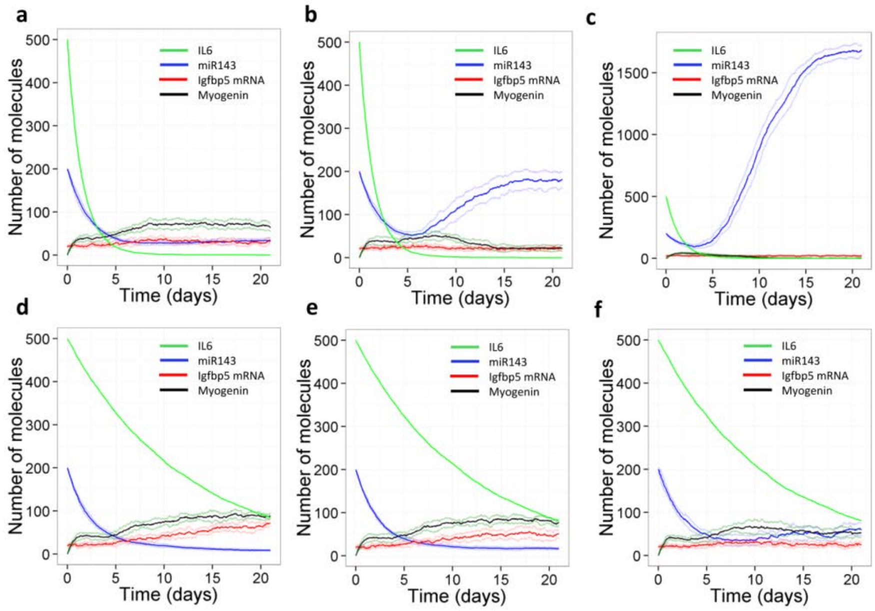

Figure S5. Simulation output from integrated model with default parameters. (**a-b**) MyoD is low, despite low Pax3 due to high levels of HoxA11. (**c**) miR-378 is low due to low levels of MyoD. (**d**) miR-143 starts to recover at day 10 and is back to basal by 21 days (not shown).

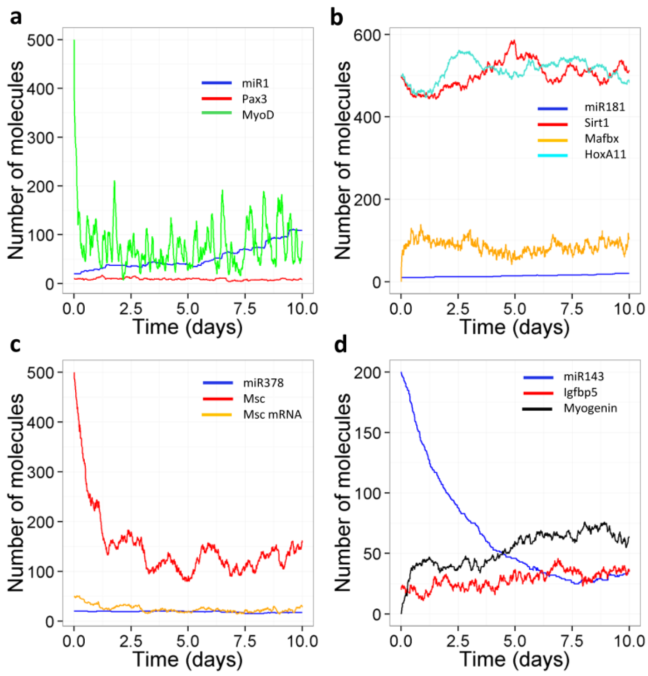

Figure S6. Simulation output from integrated model with high levels of miR-181. (**a-b**) MyoD and Mafbx is high as Sirt1 and HoxA11 protein decline. (**c**) miR-378 starts to increase due to higher levels of MyoD. (**d**) miR-143 starts to recover at day 10 and is back to basal by 21 days (not shown). (**a-d**) miR181=1000, $k_{synmiR181}$=6e-4 s^{-1}.

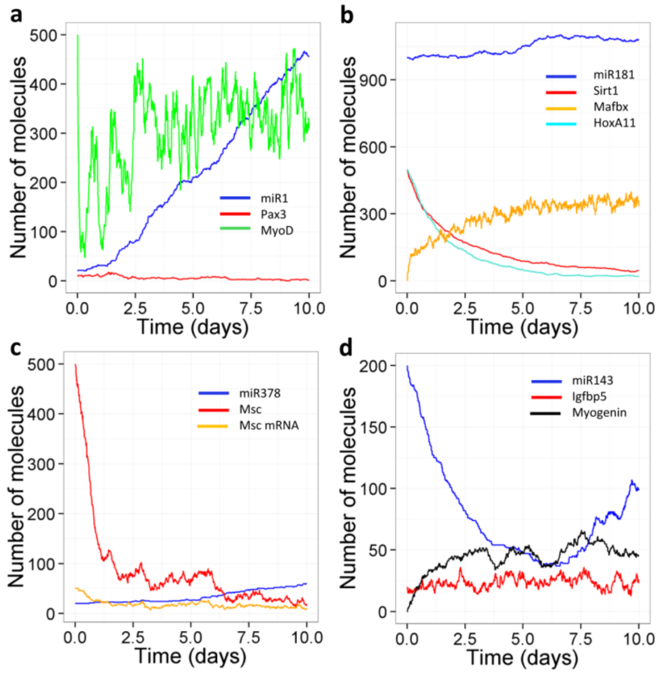

Figure S7. Simulation output from integrated model with high levels of miR-378. (**a-b**) MyoD and Mafbx are low as Sirt1 and HoxA11 protein levels are high when miR-181 is low. (**c**) Higher levels of miR-378 leads to lower levels of Msc. (**d**) Output of miR-143 components is not affected. (**a-d**) miR181=10, $k_{synmiR181}$=6e-6 s^{-1}, miR378=200, $k_{synmiR378}$=0.001 s^{-1}.

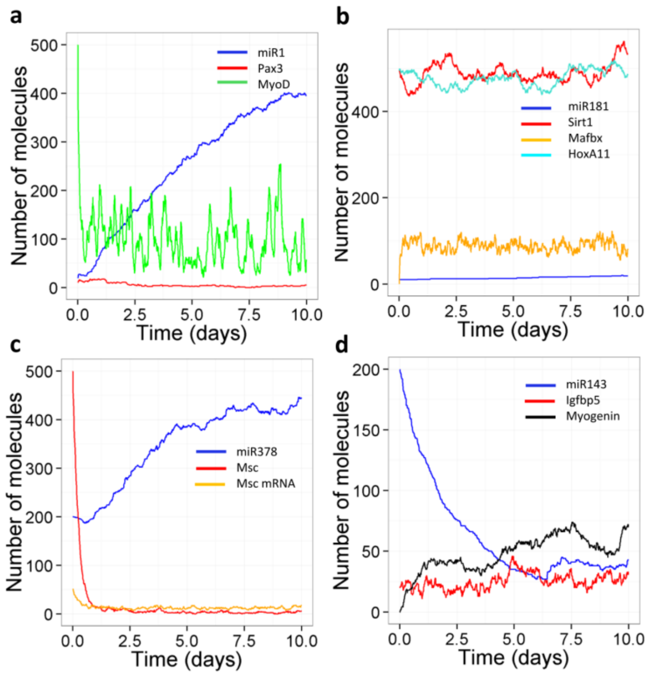

Figure S8. Simulation output from integrated model with high levels of miR-181 and miR-378. (**a-b**) MyoD and Mafbx are high as Sirt1 and HoxA11 protein levels are low when miR-181 is high. (**c**) Higher levels of MyoD lead to a further increase in miR-378 and lower levels of Msc. (**d**) Output of miR-143 components is not affected. (**a-d**) (miR181=1000, $k_{synmiR181}$=6e-4 s^{-1}, miR378=200, $k_{synmiR378}$=0.001 s^{-1}).

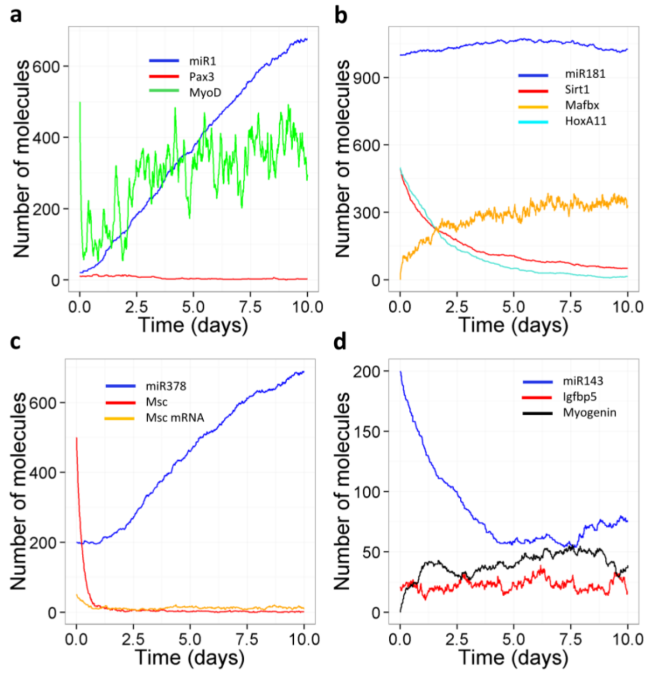

Figure S9. Simulation output from integrated model with high levels of Pax3 and low miR-181 and miR-378. (**a-b**) MyoD and Mafbx are low as Pax3, Sirt1 and HoxA11 protein levels are high. (**c**) Msc levels are high due to low levels of miR-378. (**d**) Output of miR-143 components is not affected. (**a-d**) Pax3= 1000, Pax3mRNA=400, $k_{synPax3}$=6.0e-5 s^{-1}, $k_{synPax3mRNA}$=0.08 molecules s^{-1}, miR181=10, $k_{synmiR181}$=6e-6 s^{-1}, miR378=20, $k_{synmiR378}$=1e-4 s^{-1}.

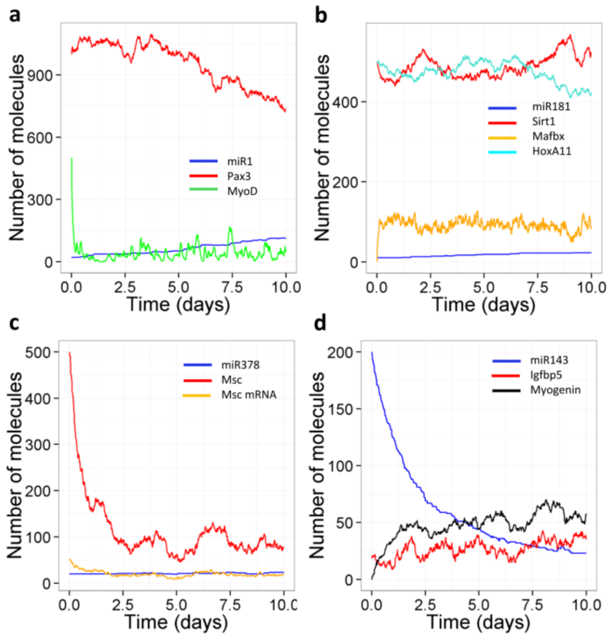

Figure S10. Simulation output from integrated model with high levels of Pax3 and high levels of miR-181 and miR-378. (**a**) miR-1 levels increase leading to inhibition of Pax3 so that MyoD levels start to increase. (**b**) Mafbx increases as high levels of miR-181 leads to decrease in Sirt1 and HoxA11 protein. (**c**) Msc levels are low due to high levels of miR-378. (**d**) Output of miR-143 components is not affected. (**a-d**) Pax3= 1000, Pax3mRNA=400, $k_{synPax3}$=6.0e-5 s^{-1}, $k_{synPax3mRNA}$=0.08 molecules s^{-1}, miR181=1000, $k_{synmiR181}$=6e-4 s^{-1}, miR378=200, $k_{synmiR378}$=0.001 s^{-1}.

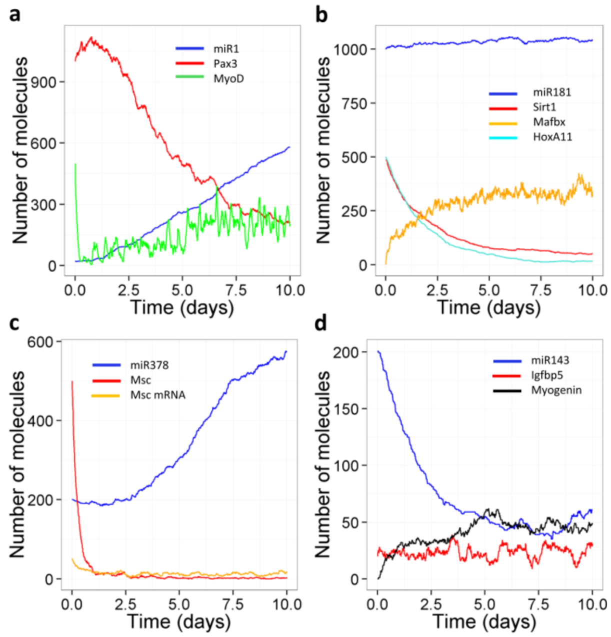

Figure S11. Simulation output from integrated model with high levels of Pax3 and high levels of miR-1, miR-181 and miR-378. (**a**) Higher miR-1 levels at the start leads to faster inhibition of Pax3 and an earlier increase in MyoD. (**b**) Components of the miR181 network are not affected by increase in miR-1. (**c**) Components of the miR378 network are not affected by increase in miR-1. (**d**) Output of miR-143 components is not affected. (**a-d**) Pax3= 1000, Pax3mRNA=400, $k_{synPax3}$=6.0e-5 s^{-1}, $k_{synPax3mRNA}$=0.08 molecules s^{-1}, miR1=1000, $k_{synmiR1}$=1e-4 s^{-1}, miR181=1000, $k_{synmiR181}$=6e-4 s^{-1}, miR378=200, $k_{synmiR378}$=0.001 s^{-1}.

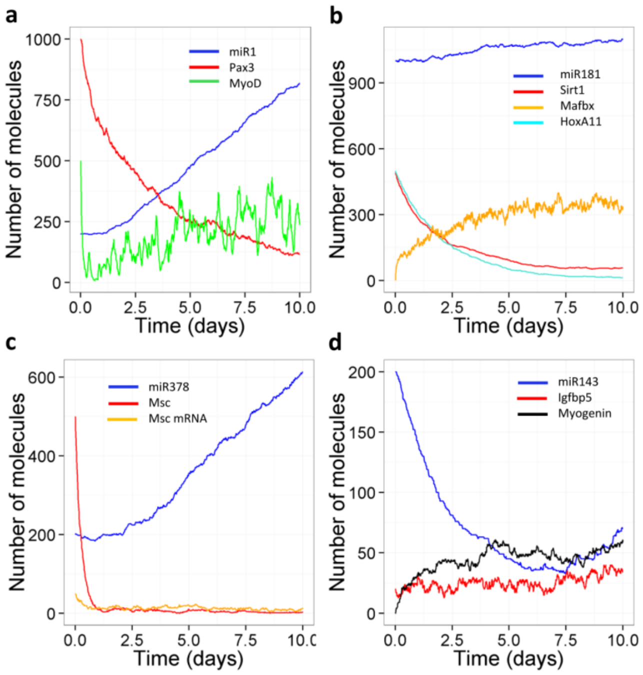

Figure S12. The effect of varying miR-143 synthesis rate when IL-6 signalling is transient or sustained in the integrated model. (**a-c**) Transient IL-6 signalling (k_{degIL6}=8e-6 s^{-1}). (**d-f**) Sustained IL-6 signalling (k_{degIL6}=1e-6 s^{-1}). (**a,d**) $k_{synmiR143}$=5e-5 s^{-1}. (**b,e**) $k_{synmiR143}$=5e-4 s^{-1} (default value). (**c,f**) $k_{synmiR143}$=5e-3 s^{-1}. The mean and standard deviation of ten individual stochastic simulations are shown.

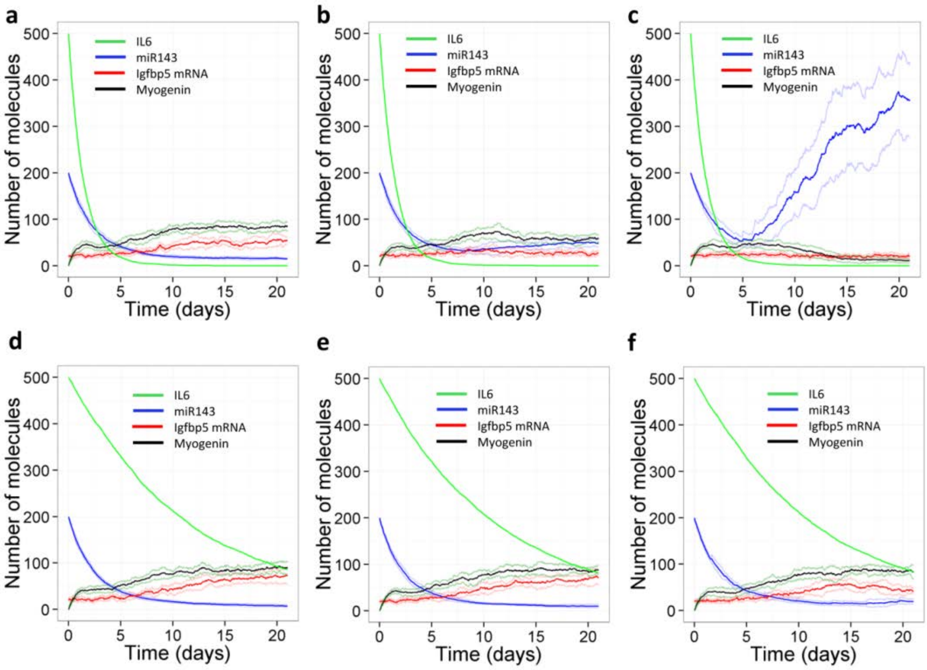

Figure S13. The effect of varying miR-143 synthesis rate when IL-6 signalling is transient or sustained with high levels of miR-181 and miR378 in the integrated model. (**a**-**c**) Transient IL-6 signalling (k_{degIL6}=8e-6 s^{-1}). (**d**-**f**) Sustained IL-6 signalling (k_{degIL6}=1e-6 s^{-1}). (**a**,**d**) $k_{synmiR143}$=5e-5 s^{-1}. (**b**,**e**) $k_{synmiR143}$=5e-4 s^{-1} (default value). (**c**,**f**) $k_{synmiR143}$=5e-3 s^{-1}. The mean and standard deviation of ten individual stochastic simulations are shown. (**a**-**f**) miR-181=1000, $k_{synmiR181}$=6e-4 s^{-1}, miR378=200, $k_{synmiR378}$=0.001 s^{-1}.

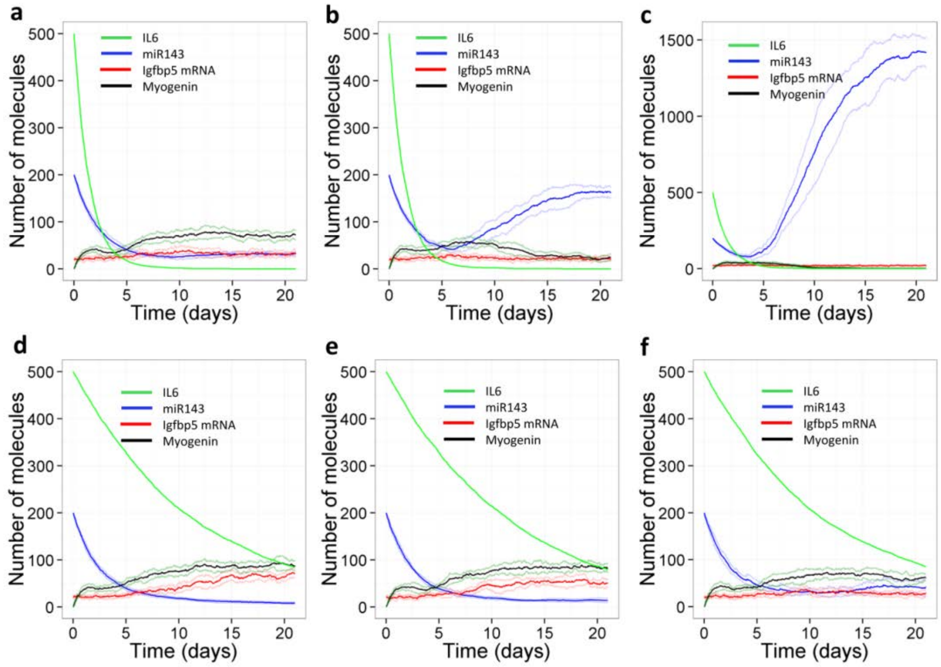

Figure S14. The effect of varying miR-378 synthesis rate on MyoD, Msc and other miRs. (**a**,**d**) Decreased miR-378 synthesis ($k_{synmiR378}$=1e-5 s^{-1}). (b,e) Default value ($k_{synmiR378}$=1e-4 s^{-1}). (**c**,**f**) Increased miR-378 synthesis ($k_{synmiR378}$=0.001 s^{-1}). The mean and standard deviation of ten individual stochastic simulations are shown.

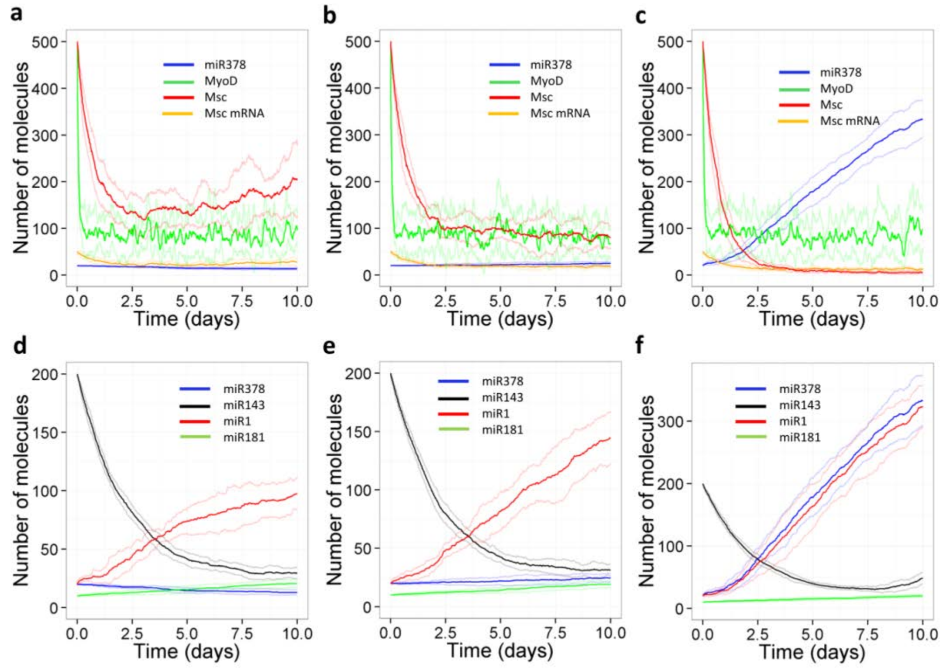

Figure S15 Network diagram of miR-1 model

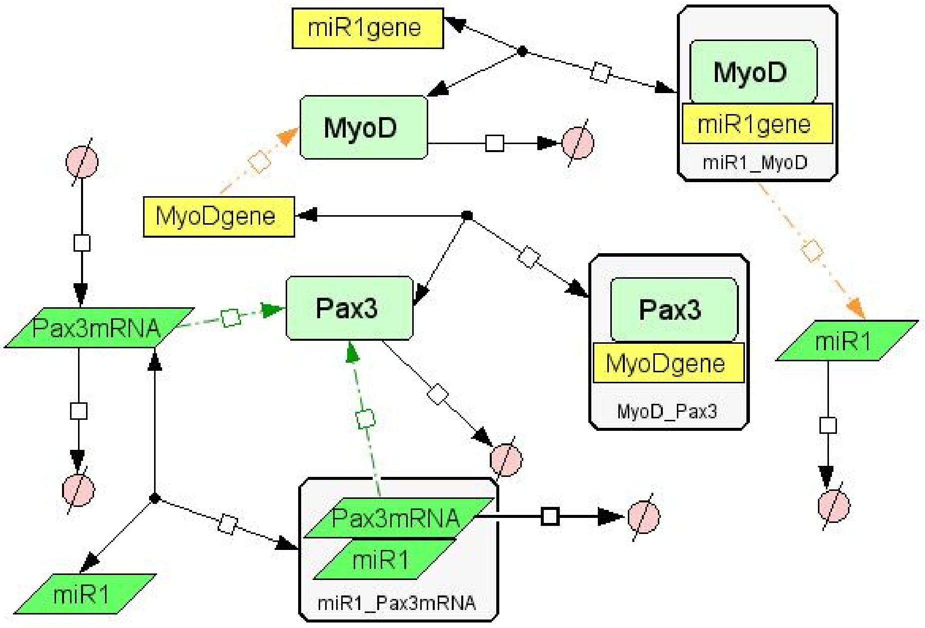

Figure S16 Network diagram of miR-181 model

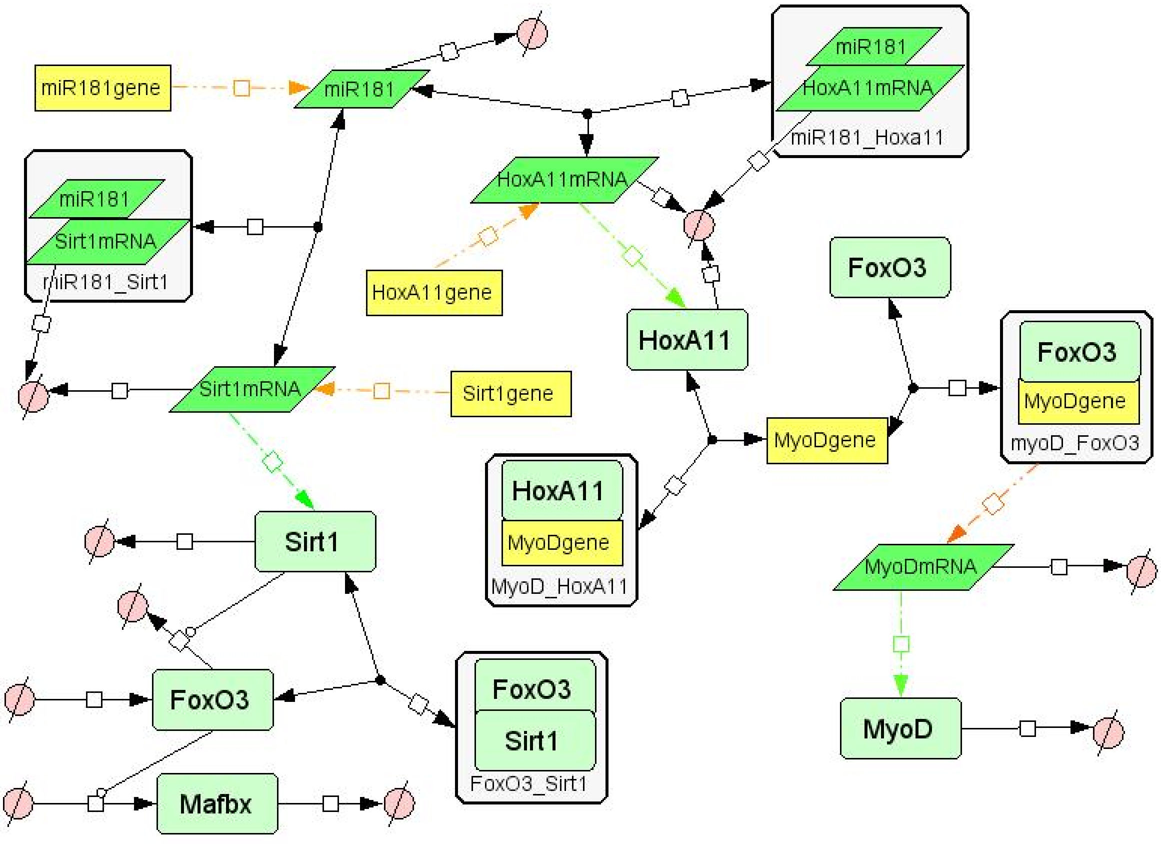

Figure S17 Network diagram of miR-378 model

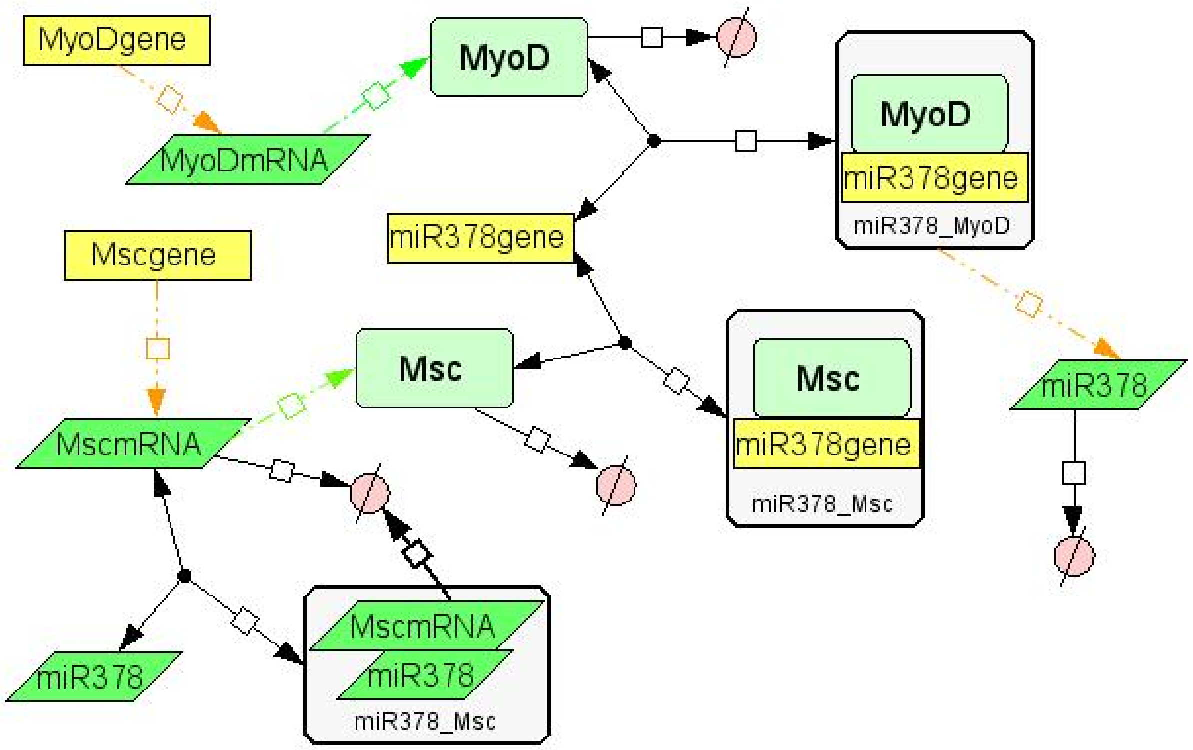

Figure S18 Network diagram of miR-143 model

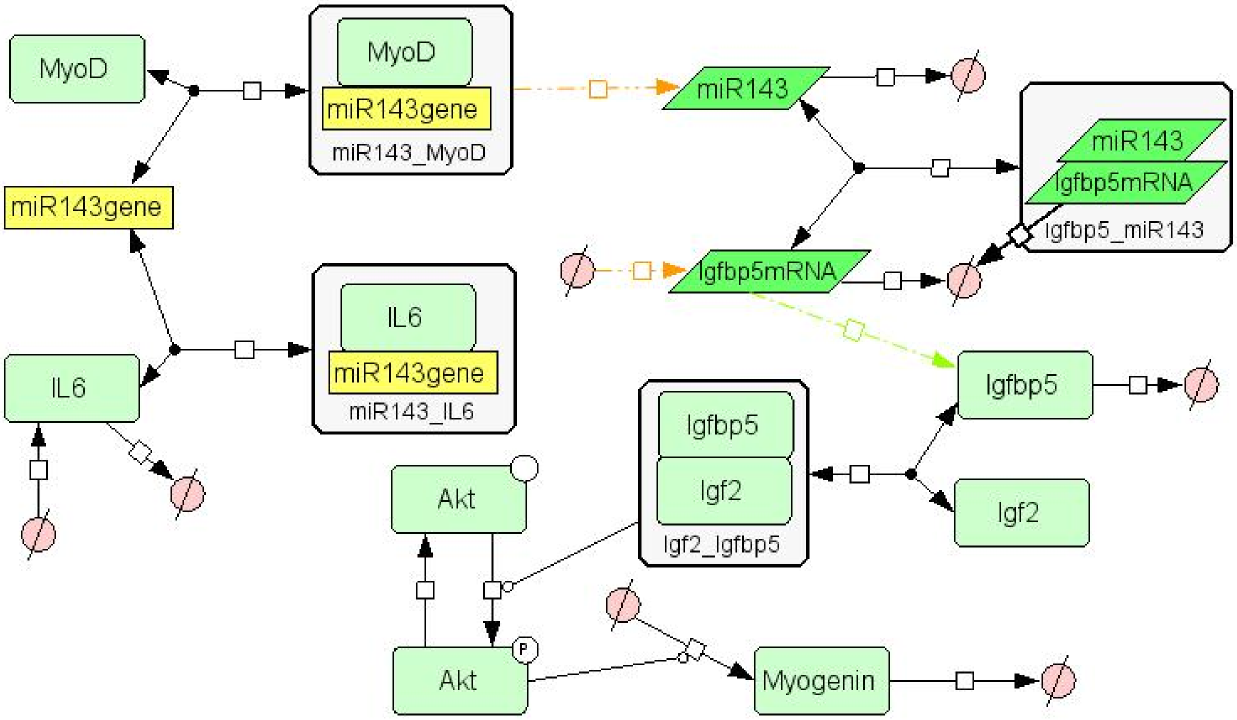

Table S1 Details of species in miR-1 model

Species ID	Description	Initial Amount
miR1	microRNA-1	20
miR1_gene	miR-1 gene	2
miR1_gene_MyoD	miR gene bound by MyoD	0
miR1_Pax3_mRNA	Pax3 mRNA bound by miR-1	0
MyoD	MyoD protein	100
MyoD_gene	MyoD gene (MYOD1)	2
MyoD_gene_Pax3	MyoD_gene bound by Pax3 to inhibit transcription	0
Pax3	Pax3 protein (PAX3)	10
Pax3_mRNA	Messenger RNA of Pax3	10

Table S2 Details of reactions in miR-1 model

Reaction ID	Reactants and products	Kinetic rate law	Parameter Value[a]
miR1_gene_MyoD_binding	miR1_gene + MyoD → miR1_gene_MyoD	$k_{binmiR1geneMyoD}$ * miR1_gene * MyoD miR_gene	1e-5 $mol^{-1}s^{-1}$
miR1_gene_MyoD_release	miR1_gene_MyoD → miR1_gene + MyoD	$k_{relmiR1geneMyoD}$ * miR1_gene_MyoD	0.001 s^{-1}
miR1_synthesis_by_MyoD	miR1_gene_MyoD → miR1_gene_MyoD + miR1	$k_{synmiR1MyoD}$ * miR1_gene_MyoD	5e-4 s^{-1}
miR1_synthesis	miR1_gene → miR1_gene + miR1	$k_{synmiR1}$ * miR1_gene	1e-8 s^{-1}
miR1_degradation	miR1 → Sink	$k_{degmiR1}$ * miR1	1.6e-6 s^{-1}
Pax3_transcription	Source → Pax3_mRNA	$k_{synPax3mRNA}$ * Source	0.002 mol s^{-1}
Pax3_mRNA_degradation	Pax3_mRNA → Sink	$k_{degPax3mRNA}$ * Pax3_mRNA	2e-4 s^{-1}
miR1_Pax3mRNA_binding	miR1 + Pax3_mRNA → miR1_Pax3_mRNA	$k_{binmiR1Pax3}$ * miR1 * Pax3_mRNA	1e-5 mol^{-1} s^{-1}
miR1_Pax3mRNA_release	miR1_Pax3_mRNA → miR1 + Pax3_mRNA	$k_{relmiR1Pax3}$ * miR1_Pax3_mRNA	0.001s^{-1}
Pax3_mRNA_degradation_by_miR1	miR1_Pax3_mRNA → miR1	$k_{degPax3mRNAmiR1}$ * miR1_Pax3_mRNA	4e-4 s^{-1}
Pax3_translation	Pax3_mRNA → Pax3_mRNA + Pax3	$k_{synPax3}$ * Pax3_mRNA	3e-5 s^{-1}
Pax3_translation2	miR1_Pax3_mRNA → miR1_Pax3_mRNA + Pax3	$k_{synPax3miR1}$ * miR1_Pax3_mRNA	5e-7 s^{-1}
Pax3_degradation	Pax3 → Sink	$k_{degPax3}$ * Pax3	2e-5 s^{-1}
MyoD_gene_ Pax3_binding	Pax3 + MyoD_gene → MyoD_gene_Pax3	$k_{binMyoDgenePax3}$ * Pax3 * MyoD_gene	1e-5 mol^{-1} s^{-1}
MyoD_gene_ Pax3_release	MyoD_gene_Pax3 → Pax3 + MyoD_gene	$k_{relMyoDgenePax3}$ * MyoD_gene_Pax3	5e-4 s^{-1}
MyoD_synthesis	MyoD_gene → MyoD_gene + MyoD	$k_{synMyoD}$ * MyoD_gene	0.1 s^{-1}
MyoD_degradation	MyoD → Sink	$k_{degMyoD}$ * MyoD	2.6e-4 s^{-1}

[a]mol=number of molecules

Table S3 Details of species in miR-181 model

Species ID	Description	Initial Amount
FoxO3	FOXO3 protein	400
FoxO3_Sirt1	FOXO3 bound by SIRT1	100
HoxA11	HOXA11 protein	500
HoxA11_mRNA	HOXA11 mRNA	100
Mafbx	Mafbx protein	0
miR181	microRNA-181	10
miR181_HoxA11_mRNA	HOXA11 mRNA bound by miR-181	0
miR181_Sirt1_mRNA	SIRT1 mRNA bound by miR-181	0
miR181_gene	miR-181 gene	2
MyoD	MyoD protein	0
MyoD_gene	MyoD gene	2
MyoD_gene_FoxO3	MyoD_gene bound by FoxO3 (activator)	0
MyoD_gene_ HoxA11	MyoD gene bound by HOXA11 (inhibitor)	0
Sirt1	SIRT1 protein	400
Sirt1_mRNA	SIRT1 mRNA	100

Table S4 Details of reactions in miR-181 model

Reaction ID	Reactants and products	Kinetic rate law	Parameter Value[a]
miR181_synthesis	miR181_gene → miR181_gene + miR181	$k_{synmiR181}$ * miR181gene	6e-6s^{-1}
miR181_degradation	miR181 → Sink	$k_{degmiR181}$ * miR181	1.6e-6 s^{-1}
Sirt1_transcription	Source → Sirt1_mRNA	$k_{synSirt1mRNA}$ * Source	0.005 mol s^{-1}
Sirt1_mRNA_degradation	Sirt1_mRNA → Sink	$k_{degSirt1mRNA}$ * Sirt1_mRNA	2e-5s^{-1}
Sirt1_translation	Sirt1_mRNA → Sirt1_mRNA + Sirt1	$k_{synSirt1}$ * Sirt1_mRNA	2e-5s^{-1}
Sirt1_degradation	Sirt1 → Sink	$k_{degSirt1}$ * Sirt1	2.4e-5s^{-1}
miR181_Sirt1mRNA_binding	miR181 + Sirt1_mRNA → miR181_Sirt1_mRNA	$k_{binmiR181Sirt1}$ * miR181 * Sirt1_mRNA	5e-5 mol^{-1} s^{-1}
miR181_Sirt1mRNA_release	miR181_Sirt1_mRNA → miR181 + Sirt1_mRNA	$k_{relmiR181Sirt1}$ * miR181_Sirt1_mRNA	0.001 s^{-1}
Sirt1_mRNA_degradation_by_miR181	miR181_Sirt1_mRNA → miR181	$k_{degSirt1mRNAmiR181}$ * miR181_Sirt1_mRNA	2e-5 s^{-1}
HoxA11_transcription	Source → HoxA11_mRNA	$k_{synHoxA11mRNA}$ * Source	0.002 mol s^{-1}
HoxA11_mRNA_degradation	HoxA11_mRNA → Sink	$k_{degHoxA11mRNA}$ * HoxA11_mRNA	2e-5 s^{-1}
miR181_HoxA11mRNA_binding	miR181 + HoxA11_mRNA → miR181_HoxA11_mRNA	$k_{binmiR181HoxA11}$ * miR181 * HoxA11_mRNA	5e-5 $mol^{-1}s^{-1}$
miR181_HoxA11mRNA_release	miR181_HoxA11_mRNA → miR181 + HoxA11_mRNA	$k_{relmiR181HoxA11}$ * miR181_HoxA11_mRNA	0.001 s^{-1}
HoxA11_mRNA_degradation_by_miR181	miR181_HoxA11_mRNA → miR181	$k_{degHoxA11mRNAmiR181}$ * miR181_HoxA11_mRNA	2e-5 s^{-1}
HoxA11_translation	HoxA11_mRNA → HoxA11_mRNA + HoxA11	$k_{synHoxA11}$ * HoxA11_mRNA	3.2e-5 s^{-1}
HoxA11_degradation	HoxA11 → Sink	$k_{degHoxA11}$ * HoxA11	6.4e-6 s^{-1}
MyoD_gene_FoxO3_binding	MyoD_gene + FoxO3 → MyoD_gene_FoxO3	$k_{binMyoDgeneFoxO3}$ * MyoD_gene * FoxO3	1e-5 $mol^{-1}s^{-1}$
MyoD_gene_FoxO3_release	MyoD_gene_FoxO3 → MyoD_gene + FoxO3	$k_{relMyoDgeneFoxO3}$ * MyoD_gene_FoxO3	0.001 s^{-1}
MyoD_transcription	MyoD_gene_FoxO3 → MyoD_gene_FoxO3 + MyoD_mRNA	$k_{synMyoDmRNA}$ * MyoD_gene_FoxO3	0.005 s^{-1}
MyoD_gene_ HoxA11_ binding	HoxA11 + MyoD_gene → MyoD_gene_ HoxA11	$k_{binMyoDgeneHoxA11}$ * HoxA11 * MyoD_gene	1e-5 $mol^{-1}s^{-1}$
MyoD_gene_ HoxA11_release	MyoD_gene_ HoxA11→ HoxA11 + MyoD_gene	$k_{relMyoDgeneHoxA11}$ * MyoD_gene_ HoxA11	5e-4 s^{-1}
MyoD_translation	MyoD_mRNA → MyoD_mRNA + MyoD	$k_{synMyoD}$ * MyoD_mRNA	0.002 s^{-1}
MyoD_mRNA_degradation	MyoD_mRNA → Sink	$k_{degMyoDmRNA}$ * MyoD_mRNA	2e-4 s^{-1}
MyoD_degradation	MyoD → Sink	$k_{degMyoD}$ * MyoD	2.6e-4 s^{-1}
FoxO3_Sirt1_binding	FoxO3 + Sirt1 → FoxO3_Sirt1	$k_{binFoxO3Sirt1}$ * FoxO3 * Sirt1	1e-5 mol^{-1} s^{-1}
FoxO3_Sirt1_release	FoxO3_Sirt1 → FoxO3 + Sirt1	$k_{relFoxO3Sirt1}$ * FoxO3_Sirt1	0.001 s^{-1}
Fox3_degradation_via_Sirt1	FoxO3_Sirt1 → Sirt1	$k_{degFoxO3Sirt1}$ * FoxO3_Sirt1	9.6e-5 s^{-1}
Fox3_degradation	FoxO3 → Sink	$k_{degFoxO3}$ * FoxO3	4.8e-5 s^{-1}
Fox3_synthesis	Source → FoxO3	$k_{synFoxO3}$ * Source	0.038 mol s^{-1}
Mafbx_synthesis	FoxO3 → FoxO3 + Mafbx	$k_{synMafbx}$ * FoxO3	1e-4 s^{-1}
Mafbx_degradation	Mafbx → Sink	$k_{degMafbx}$ * Mafbx	2e-4 s^{-1}

[a]mol=number of molecules

Table S5 Details of species in miR-378 model

Species ID	Description	Initial Amount
miR378	microRNA-378	20
miR378_gene	miR-378 gene	2
miR378_gene_MyoD	miR-378 gene bound by MyoD (activator)	0
miR378_gene_Msc	miR-378 gene bound by Msc (inhibitor)	0
miR378_Msc_mRNA	Msc mRNA bound by miR378	0
MyoD	MyoD protein	500
MyoD_gene	MyoD gene	2
MyoD_mRNA	MyoD mRNA	20
Msc	Musculin protein (also known as MyoR)	500
Msc_gene	Msc gene	2
Msc_mRNA	Msc mRNA	50

Table S6 Details of reactions in miR-378 model

Reaction ID	Reactants and products	Kinetic rate law	Parameter Value[a]
miR378_gene_MyoD_binding	miR378_gene + MyoD → miR378_gene_MyoD	$k_{binmiR378geneMyoD}$ * miR378_gene * MyoD	1e-5 $mol^{-1} s^{-1}$
miR_gene_MyoD_release	miR378_gene_MyoD → miR378_gene + MyoD	$k_{relmiR378geneMyoD}$ * miR378_gene_MyoD	0.001 s^{-1}
miR378_synthesis	miR378_gene_MyoD → miR378_gene_MyoD + miR378	$k_{synmiR378}$ * miR378_gene_MyoD	1e-4 s^{-1}
miR378_degradation	miR378 → Sink	$k_{degmiR378}$ * miR378	1.6e-6 s^{-1}
MyoD_transcription	MyoD_gene → MyoD_gene + MyoD_mRNA	$k_{synMyoDmRNA}$ * MyoD_gene	0.005 s^{-1}
MyoD_mRNA_degradation	MyoD_mRNA → Sink	$k_{degMyoDmRNA}$ * MyoD_mRNA	2e-4 s^{-1}
MyoD_translation	MyoD_mRNA → MyoD_mRNA + MyoD	$k_{synMyoD}$ * MyoD_mRNA	0.002 s^{-1}
MyoD_degradation	MyoD → Sink	$k_{degMyoD}$ * MyoD	2.6e-4 s^{-1}
Msc_transcription	Msc_gene → Msc_gene + Msc_mRNA	$k_{synMscmRNA}$ * Msc_gene	2.5e-4 s^{-1}
Msc_translation	Msc_mRNA → Msc_mRNA + Msc	k_{synMsc} * Msc_mRNA	5e-4 s^{-1}
Msc_mRNA_degradation	Msc_mRNA → Sink	$k_{degMscmRNA}$ * Msc_mRNA	1e-5 s^{-1}
miR378_Msc_binding	miR378 + Msc_mRNA → miR378_Msc_mRNA	$k_{binmiR378MscmRNA}$ * miR378 * Msc_mRNA	1e-4 $mol^{-1} s^{-1}$
miR378_Msc_release	miR378_Msc_mRNA → miR378 + Msc_mRNA	$k_{relmiR378MscmRNA}$ * miR378_Msc_mRNA	0.001 s^{-1}
Msc_mRNA_degradation_by_miR378	miR378_Msc_mRNA → miR378	$k_{degMscmRNAmiR378}$ * miR378_Msc_mRNA	4e-5 s^{-1}
miR378_gene_Msc_binding	miR378_gene + Msc → miR378_gene_Msc	$k_{binmiR378geneMsc}$ * miR378_gene * Msc	5e-5 $mol^{-1} s^{-1}$
miR378_gene_Msc_release	miR378_gene_Msc → miR378_gene + Msc	$k_{relmiR378geneMsc}$ * miR378_gene_Msc	0.001 s^{-1}
Msc_degradation	Msc → Sink	k_{degMsc} * Msc	5e-5 s^{-1}

[a]mol=number of molecules

Table S7 Details of species in miR-143 model

Species ID	Description	Initial Amount
Akt	Akt protein	100
Akt_P	Phosphorylated Akt	0
Igf2	Igf2 protein	500
Igf2_Igfbp5	Igf2 bound by Igfbp5	0
Igfbp5	Igfbp5 protein	100
Igfbp5_mRNA	Igfbp5 mRNA	20
Igfbp5_mRNA_miR143	Igfbp5 mRNA bound by miR143	0
IL6	IL6 protein	500
miR143	microRNA-143	200
miR143_gene	miR-143 gene	2
miR143_gene_IL6	miR143 bound by a TF activated by IL6	0
miR143_gene_MyoD	miR-143 gene bound by MyoD	0
MyoD	MyoD protein	500
Myogenin	Myogenin protein	0

Table S8 Details of reactions in miR-143 model

Reaction ID	Reactants and products	Kinetic rate law	Parameter Value[a]
miR143_gene_MyoD_binding	miR143_gene + MyoD → miR143_gene_MyoD	$k_{binmiR143geneMyoD}$ * miR143_gene * MyoD	1e-5 $mol^{-1}s^{-1}$
miR143_gene_MyoD_release	miR143_gene_MyoD → miR143_gene + MyoD	$k_{relmiR143geneMyoD}$ * miR143_gene_MyoD	0.001 s^{-1}
miR143_synthesis	miR143_gene_MyoD → miR143_gene_MyoD + miR143	$k_{synmiR143}$ * miR143_gene_MyoD	5e-4 s^{-1}
miR143_degradation	miR143 → Sink	$k_{degmiR143}$ * miR143	5e-6 s^{-1}
IL6_degradation	IL6 → Sink	k_{degIL6} * IL6	8e-6 s^{-1}
miR143_gene_IL6_binding	miR143_gene + IL6 → miR143_gene_IL6	$k_{binmiR143geneIL6}$ * miR143_gene * IL6	0.001 $mol^{-1}s^{-1}$
miR143_gene_IL6_release	miR143_gene_IL6 → miR143_gene + IL6	$k_{relmiR143geneIL6}$ * miR143_gene_IL6	5e-4 s^{-1}
Igfbp5_transcription	Source → Igfbp5_mRNA	$k_{synIgfbp5mRNA}$ * Source	0.001 mol s^{-1}
Igfbp5_mRNA_degradation	Igfbp5_mRNA → Sink	$k_{degIgfbp5mRNA}$ * Igfbp5_mRNA	1e-5 s^{-1}
Igfbp5_mRNA_miR143_ binding	Igfbp5_mRNA + miR143 → Igfbp5_mRNA_miR143	$k_{binmiR143Igfbp5mRNA}$ * Igfbp5_mRNA * miR143	1e-4 $mol^{-1}s^{-1}$
Igfbp5_mRNA_miR143_ release	Igfbp5_mRNA_miR143 → Igfbp5_mRNA + miR143	$k_{relmiR143Igfbp5mRNA}$ * Igfbp5_mRNA_miR143	0.001 s^{-1}
Igfbp5_mRNA_degradation_by _miR143	Igfbp5_mRNA_miR143 → miR143	$k_{degIgfbp5mRNAmiR143}$ * Igfbp5_mRNA_miR143	5e-5 s^{-1}
Igfbp5_translation	Igfbp5_mRNA → Igfbp5_mRNA + Igfbp5	$k_{synIgfbp5}$ * Igfbp5_mRNA	8e-5 s^{-1}
Igfbp5_degradation	Igfbp5 → Sink	$k_{degIgfbp5}$ * Igfbp5	5e-5 s^{-1}
Igf2_Igfbp5_binding	Igf2 + Igfbp5 →Igf2_Igfbp5	$k_{binIgf2Igfbp5}$ * Igf2 * Igfbp5	5e-6 $mol^{-1}s^{-1}$
Igf2_Igfbp5_release	Igf2_Igfbp5 →Igf2 + Igfbp5	$k_{relIgf2Igfbp5}$ * Igf2_Igfbp5	0.001 s^{-1}
Akt_phosphorylation	Igf2_Igfbp5 + Akt → Igf2_Igfbp5 + Akt_P	$k_{phosAkt}$ * Igf2_Igfbp5 * Akt	5e-4$mol^{-1}s^{-1}$
Akt_dephosphorylation	Akt_P → Akt	$k_{dephosAkt}$ * Akt_P	0.01 s^{-1}
Myogenin_synthesis	Akt_P → Akt_P + Myogenin	$k_{synMyogenin}$ * Akt_P	1e-5s^{-1}
Myogenin_degradation	Myogenin → Sink	$k_{degMyogenin}$ * Myogenin	1e-5 s^{-1}

[a]mol=number of molecules

Table S9 Database Terms of Model Species

**Model name**	Approved gene name	Database term
Akt	AKT1	HGNC:391
FoxO3	FOXO3	HGNC:3821
HoxA11	HOXA11	HGNC:5101
Igf2	IGF2	HGNC:5466
Igfbp5	IGFBP5	HGNC:5474
IL6	IL6	HGNC:6018
Mafbx	FBX032	HGNC:16731
miR1	MIR1-1	HGNC:31499
miR143	MIR143	HGNC:31530
miR181	MIR181A1	HGNC:31590
mir378	MIR378A	HGNC:31871
MyoD	MYOD1	HGNC:7611
Myogenin	MYOG	HGNC:7612
Msc	MSC	HGNC:7321
Pax3	PAX3	HGNC:8617
Sirt1	SIRT1	HGNC:14929

References

1 Goljanek-Whysall, K. *et al.* MicroRNA regulation of the paired-box transcription factor Pax3 confers robustness to developmental timing of myogenesis. *Proc. Natl. Acad. Sci. U. S. A.* **108**, 11936-11941, doi:10.1073/pnas.1105362108 (2011).

2 Naguibneva, I. *et al.* The microRNA miR-181 targets the homeobox protein Hox-A11 during mammalian myoblast differentiation. *Nat. Cell Biol.* **8**, 278-284, doi:10.1038/ncb1373 (2006).

3 Soriano-Arroquia, A., House, L., Tregilgas, L., Canty-Laird, E. & Goljanek-Whysall, K. The functional consequences of age-related changes in microRNA expression in skeletal muscle. *Biogerontology* **17**, 641-654, doi:10.1007/s10522-016-9638-8 (2016).
